# The Distribution of Coumarins and Furanocoumarins in *Citrus* Species Closely Matches *Citrus* Phylogeny and Reflects the Organization of Biosynthetic Pathways

**DOI:** 10.1371/journal.pone.0142757

**Published:** 2015-11-11

**Authors:** Audray Dugrand-Judek, Alexandre Olry, Alain Hehn, Gilles Costantino, Patrick Ollitrault, Yann Froelicher, Frédéric Bourgaud

**Affiliations:** 1 Université de Lorraine, UMR 1121 Laboratoire Agronomie et Environnement Nancy-Colmar, 2 avenue de la forêt de Haye, TSA 40602, 54518, Vandœuvre-lès-Nancy, France; 2 INRA, UMR 1121 Université de Lorraine, UMR 1121 Laboratoire Agronomie et Environnement Nancy-Colmar, 2 avenue de la forêt de Haye, TSA 40602, 54518, Vandœuvre-lès-Nancy, France; 3 INRA, UMR AGAP, Station INRA, F-20230, San Giuliano, France; 4 Centro de Protección Vegetal y Biotecnología, Instituto Valenciano de Investigaciones Agrarias (Ivia), 46113 Moncada, Valencia, Spain; 5 CIRAD, UMR AGAP, Station de Roujol, 97170 Petit-Bourg, Guadeloupe, France; 6 CIRAD, UMR AGAP, Station INRA, F-20230, San Giuliano, France; USDA/ARS, UNITED STATES

## Abstract

*Citrus* plants are able to produce defense compounds such as coumarins and furanocoumarins to cope with herbivorous insects and pathogens. In humans, these chemical compounds are strong photosensitizers and can interact with medications, leading to the “grapefruit juice effect”. Removing coumarins and furanocoumarins from food and cosmetics imply additional costs and might alter product quality. Thus, the selection of *Citrus* cultivars displaying low coumarin and furanocoumarin contents constitutes a valuable alternative. In this study, we performed ultra-performance liquid chromatography coupled with mass spectrometry analyses to determine the contents of these compounds within the peel and the pulp of 61 *Citrus* species representative of the genetic diversity all *Citrus*. Generally, *Citrus* peel contains larger diversity and higher concentrations of coumarin/furanocoumarin than the pulp of the same fruits. According to the chemotypes found in the peel, *Citrus* species can be separated into 4 groups that correspond to the 4 ancestral taxa (pummelos, mandarins, citrons and papedas) and extended with their respective secondary species descendants. Three of the 4 ancestral taxa (pummelos, citrons and papedas) synthesize high amounts of these compounds, whereas mandarins appear practically devoid of them. Additionally, all ancestral taxa and their hybrids are logically organized according to the coumarin and furanocoumarin pathways described in the literature. This organization allows hypotheses to be drawn regarding the biosynthetic origin of compounds for which the biogenesis remains unresolved. Determining coumarin and furanocoumarin contents is also helpful for hypothesizing the origin of *Citrus* species for which the phylogeny is presently not firmly established. Finally, this work also notes favorable hybridization schemes that will lead to low coumarin and furanocoumarin contents, and we propose to select mandarins and Ichang papeda as *Citrus* varieties for use in creating species devoid of these toxic compounds in future breeding programs.

## Introduction

Citrus originate from the tropical and subtropical regions of Southeast Asia and their culture was initiated in India and China during the first millenary BC [1]. Currently, citrus are cultivated between the latitudes 40°N and 40°S in tropical and subtropical regions [2]. In 2013, citrus crops represented the most important fruit produced in the world, with more than 135 million tons [3]. Citrus belong to the Rutaceae family, which is composed of approximately 160 genera and 1900 species [4]; true citrus belong to the Aurantioideae subfamily, the Citreae tribe and the Citrineae subtribe. True citrus include 6 genera: *Citrus*, *Fortunella*, *Poncirus*, *Microcitrus*, *Eremocitrus* and *Clymenia* [5]. Citrus taxonomy remains controversial due to a long cultivation history, complex reproductive biology and somatic bud mutation. Swingle and Reece (1967) [5] and Tanaka (1977) [6], which recognize 16 and 162 species, respectively, remain the two major classification systems currently used. However, this last decade, molecular analyses have provided decisive information regarding *Citrus* domestication and the relations between various cultivated species of *Citrus* [7–14]. Four ancestral taxa, *Citrus medica* L. (citron), *Citrus reticulata* Blanco (mandarin), *Citrus maxima* (Burm.) Merr. (pummelo), and *Citrus micrantha* Wester (papeda), have been identified as the ancestors of all cultivated Citrus [8,10,12,14]. Among the four basic horticultural groups, no evidence of interspecific introgression was found in pummelos, citrons, and *C*. *micrantha*. In contrast, evidence of introgression by *C*. *maxima* was found in sweet mandarin by sequencing and resequencing [13,14]. However, ‘Cleopatra’, ‘Sunki’ and ‘Shekwasha’ mandarin, which share the acidic mandarin cytoplasm defined by Froelicher *et al*. (2011) [10] from mitochondrial data and confirmed by Carbonell-Caballero *et al*. (2015) [15] with chloroplast data, present no evidence of introgression [14]. The secondary species *Citrus sinensis* (L.) Osb. (sweet orange), *Citrus aurantium* L. (sour orange), *Citrus paradisi* Macf. (grapefruit), *Citrus limon* (L.) Burm. (lemon), and *Citrus aurantifolia* (Christm.) Swing. (lime) arose from the hybridization of the 4 ancestral taxa [8,10,12,14]. In fact, *C*. *sinensis*, *C*. *aurantium*, *C*. *paradisi*, and tangors had a 2 ancestral taxa admixture structure (*C*. *reticulata* and *C*. *maxima*) with variable contributions. The origins of *C*. *limon* and *C*. *aurantifolia* (Swingle and Reece taxonomy) are more complex and come from 2 or 3 taxa hybridization. Similarly, *C*. *aurantifolia* originates from different taxa hybridization (2 to 4 taxa) composed systematically of citron and papeda taxa. *Citrus amblycarpa* secondary species displayed approximately 50% contributions from *C*. *reticulata* and *C*. *micrantha* [14].

**Fig 1:**
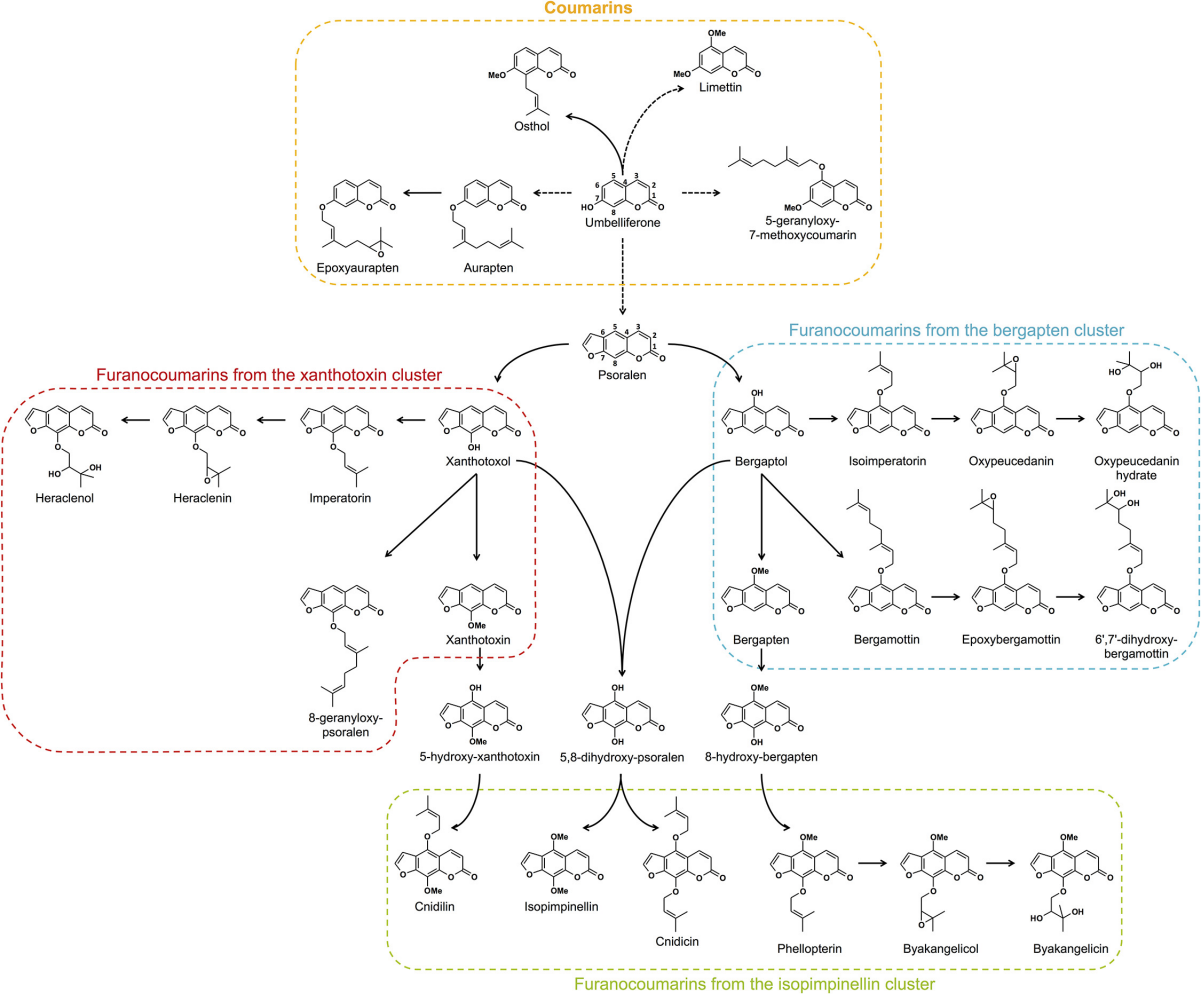
Schematic representation of the coumarin and furanocoumarin pathways. Solid lines indicate direct enzymatic steps; dashed lines represent multiple enzymatic steps. Coumarins are framed in orange, furanocoumarins of the bergapten cluster are framed in blue, furanocoumarins of the xanthotoxin cluster are framed in red, and furanocoumarins of the isopimpinellin cluster are framed in green.

Coumarins (benzo-α-pyrones) constitute a class of secondary metabolites commonly found in higher plants [16]. In a limited number of plant families, such as Rutaceae, Moraceae, Apiaceae and Fabaceae, umbelliferone (7-hydroxycoumarin), a ubiquitous coumarin of higher plants, can undergo subsequent biochemical modifications corresponding to a prenylation step at C6 or C8 followed by the closure of a furan ring. These modifications generate furanocoumarins, a new subclass of compounds involved in plant defense against pathogens [16] and, more generally, in plant environmental adaptation [16,17]. Citrus, as members of the Rutaceae, can synthesize both coumarins and furanocoumarins and are rich in dimethylallylated and/or geranylated compounds (see Fig 1) such as bergamottin (5-geranyloxypsoralen), aurapten (7-geranyloxycoumarin) or imperatorin (8-dimethylallyloxypsoralen).

In parallel to their ecological functions in plants, coumarins and furanocoumarins can be deleterious for humans. These compounds are potential photosensitizers that can cause severe phytophotodermatitis after either skin contact [18] or ingestion [19] followed by sun UV exposure. This photosensitization property is a notable problem with citrus essential oils such as bergamot oil because they are extensively used in perfumes and cosmetics. In addition, furanocoumarin monomers [20] and dimers such as paradisins [21] are known to inhibit cytochrome P450 (CYP) from different families [22,23]. Notably, interactions between furanocoumarins and human CYP3A4, a CYP found in intestinal enterocytes and in liver hepatocytes, have generated many concerns because this P450 (i) represents half of all human P450 enzyme pools and (ii) plays a major role in the metabolism of drugs altered by oxidation [24]. CYP3A4 furanocoumarin-mediated inhibition increases the bioavailability of possibly more than 85 medicines, among which 43 can lead to severe adverse effects, such as heart rhythm disorders or respiratory depression [25]. This interaction was first discovered with grapefruit juice consumption in the beginning of the 1990s [26] and is often called the "grapefruit juice effect". Since then, grapefruit consumption steadily declined from approximately 2.4 million tons in 1991 to approximately 1.75 million tons in 2009 in the developed countries aware of this effect [27]. Yet, citrus fruits also display beneficial effects on health, such as antioxidant properties [28], neuroprotective effects [29] or the reduction of cardiovascular risk [30].

In relation to the undesirable effects of furanocoumarins in humans, different studies have aimed at removing these toxicants from grapefruit juices or citrus essential oils. For example, furanocoumarins can be removed by different extraction and absorption processes [31], degraded by heat treatment [32] or ultraviolet irradiation [33], or removed by the use of autoclaved fungi [34]. However, these treatments imply additional costs and might alter the qualities of grapefruit juice or essential oils. Because the furanocoumarin content in plants is determined by the occurrence of specialized genes [35–38] and is therefore highly heritable, an alternative solution for lowering the furanocoumarin concentration in citrus would consist of developing new breeding programs that would allow access to new citrus varieties with low or zero furanocoumarin content.

Thus far, several studies have attempted to identify and quantitate coumarins and furanocoumarins in citrus tissue [39–42]. However, none of these studies was sufficiently comprehensive regarding the number of chemical compounds and/or citrus varieties that were studied. Thus, a comprehensive study regarding coumarin and furanocoumarin distribution among citrus varieties in terms of both the diversity of chemicals and quantities of compounds synthesized is necessary for building up future breeding projects.

Because our objective was to perform quantitative analyses of already known coumarins and furanocoumarins in citrus, we limited our study to the identification and quantitation of compounds for which authentic standards were available. Under these premises, the present study reports the chemical diversity represented by 27 coumarins and furanocoumarins in 61 *Citrus* varieties from 21 different species (according to Swingle’s classification) that are representative of the genetic diversity of all *Citrus*. Our study focused on the sole *Citrus* genus, as it is the largest of the 6 true citrus genera in terms of species number, the most cultivated and the most used by the food and perfume industries. The 4 ancestral taxa were represented by 7 pummelos, 11 mandarins (8 sweet mandarins and 3 acidic mandarins), 3 citrons and 3 papedas. Secondary species were also investigated, with 4 sweet oranges, 4 sour oranges, 3 grapefruits, 4 small mandarin hybrids, 7 lemons, 10 limes, 1 bergamot and 4 other hybrids: Halimii (Mountain), Yuzu, Latipes (Khasi) and Amblycarpa (Nasnaran mandarin). Both citrus peel and pulp were analyzed because the pulp is used for human consumption and because the peel can be used as a flavoring or fragrance ingredient in food and perfumes.

The coumarin and furanocoumarin contents found in citrus peel and pulp samples are compared, and the chemical diversity is discussed with respect to the genetic diversity of the studied varieties and to the organization of the biosynthetic pathways. With reference to the *Citrus* diversity obtained from molecular markers, we highlight the genetic crossings that may have resulted in low coumarin and furanocoumarin content varieties. Finally, the *Citrus* varieties with low coumarin and furanocoumarin content identified in this study will constitute invaluable genetic resources for future breeding programs, promoting new citrus species devoid of these toxic compounds.

## Materials and Methods

### Plant material

Citrus fruits (except *C*. *micrantha*) were harvested at the *Citrus* Biological Resource Center (CRB CITRUS, INRA-CIRAD, France) at San Giuliano (Corsica, France). *C*. *micrantha* fruits were harvested at Bachès nursery located in Eus (France). This study included 61 *Citrus* varieties representative of the *Citrus* diversity described in Table 1 and in S1 Table. For each *Citrus* variety, 5 fruits were harvested from the tree.

**Table 1:** List of the accessions investigated in the study and their phylogenetic constitution.

	Swingle's classification system	Phylogenetic constitution	Number of accessions
**Horticultural group of ancestral species**			
Papeda	*C*. *micrantha* Wester	*C*. *micrantha*	2
Citron	*C*. *medica* L.	*C*. *medica*	3
Pummelo	*C*. *maxima* (Burm.) Merr.	*C*. *maxima*	7
Mandarin	*C*. *reticulata* Blanco	*C*. *reticulata*	11
**Horticultural group of secondary species**			
Small mandarin hybrid	*C*. *reticulata* × *C*. *sinensis*	*C*. *reticulata* x ((*C*. *maxima x C*. *reticulata*) *x C*. *reticulata*)	1
	*C*. *reticulata* × *C*. *sinensis*?	*C*. *reticulata* x ((*C*. *maxima x C*. *reticulata*) *x C*. *reticulata*)?	1
	*C*. *reticulata* Blanco	?	1
	(*C*. *reticulata* × *C*. *sinensis*) × *C*. *tangerina*	((*C*. *reticulata* x ((*C*. *maxima x C*. *reticulata*) *x C*. *reticulata*))) x (C. reticulata x (C. maxima x ((C. maxima x C. reticulata) x C. reticulata)))	1
Sweet orange	*C*. *sinensis* (L.) Osbeck	(*C*. *maxima x C*. *reticulata*) *x C*. *reticulata*	4
Grapefruit	*C*. *paradisi* Macf.	*C*. *maxima* x ((*C*. *maxima x C*. *reticulata*) *x C*. *reticulata*)	3
Sour orange	*C*. *aurantium* L.	*C*. *maxima x C*. *reticulata*	4
Lemon	*C*. *limon* (L.) Burm. f.	*(C*. *maxima x C*. *reticulata)* x *C*. *medica*	2
		*(C*. *maxima x C*. *reticulata)* x *C*. *medica* ***or*** *((C*. *maxima x C*. *reticulata)x C*. *reticulata) x C*. *medica*	1
		*C*. *reticulata* x *C*. *medica*	4
Lime	*C*. *aurantifolia* (Christm.) Swing.	*C*. *micrantha x C*. *medica*	4
		2 (*C*. *micrantha x C*. *medica*) x *C*. *medica*	1
		((*C*. *maxima x C*. *reticulata*) x *C*. *medica*) x 2 (*C*. *micrantha x C*. *medica*)	2
		(*C*. *maxima x C*. *reticulata*) x *C*. *medica*	2
Bergamot	*C*. *aurantifolia* var Bergamia	((*C*. *maxima* x *C*. *reticulata*) x *C*. *medica*) x (*C*. *maxima* x *C*. *reticulata*)	1
Amblycarpa	*C*. *reticulata* Blanco hybrid	*C*. *micrantha x C*. *reticulata*	1
Mandarin × citron hybrid	*C*. *reticulata* Blanco var. austera Swing. x *C*. *medica* L.	*C*. *reticulata* x *C*. *medica*	1
Ichang	*C*. *ichangensis* Swing.	?	1
Halimii	*C*. *halimii* B.C. Stone (Tan. Class.)	?	1
Yuzu	*C*. *ichang austera* ex. Tan. hybrid	?	1
Latipes	*C*. *latipes* (Swing.) Tan.	?	1

### Sample preparation

This study was performed on citrus peel and pulp samples. All fruits were harvested and sampled as described previously [39]. Briefly, citrus peel samples consisted of the flavedo and albedo tissues, without exceeding 4 mm for the albedo part, as its thickness greatly varies from one species to the other. Citrus pulp samples only consisted of the juice vesicles, without the walls separating the segments, which were excluded. The only difference with the citrus peel samples is that the citrus pulp was ground for only 2 minutes in the ball mill. Fresh samples were weighed, frozen in liquid nitrogen and stored in a freezer at -80°C until extraction was performed.

### Extraction of compounds

The extraction of the compounds from the peel and pulp samples was identical to the method described by Dugrand *et al*. (2013) [39]. In brief, frozen samples were lyophilized and ground in a ball mill. Two extractions of the compounds were conducted in 80:20 methanol HPLC-grade/water solutions. The citrus extracts obtained were evaporated and then resuspended in a 75:25 methanol HPLC-grade/ultrapure water solution.

### UPLC-MS analyses and data processing

UPLC-MS analyses and data processing were performed with the same equipment and methods described by Dugrand *et al*. (2013) [39]. In short, the UPLC separation of the coumarins and furanocoumarins was performed on a C18 reversed-phase column thermostated at 40°C. The mobile phase consisted of 0.1% formic acid in ultrapure water (A) and 0.1% formic acid in methanol (B). The elution gradient was (A:B; v/v): 90:10 at 0 min, 80:20 at 0.74 min, 40:60 at 5.88 min, 10:90 at 10 min, 0:100 between 12 and 16 min, and 90:10 from 16.01 to 20 min. The total analysis lasted 20 min. The flow rate was set at 0.2 mL/min and the injection volume was 3 μL. Concerning the MS system, we used a dual ion source (DUIS). The inlet, desolvatation line and heating block temperatures were respectively set at 350, 250 and 400°C. The voltage of the capillary was set at 4.5 kV. The detection of the compounds was performed in single ion monitoring mode (SIM). Data were acquired and processed on LabSolution software version 5.52 sp2 (Shimadzu).

### Method validation

Method validation has been performed for citrus peel samples in our previous work [39] but has not been performed for citrus pulp samples. Full validation of a method includes checking the linearity, limits of detection (LOD) and quantitation (LOQ), specificity, accuracy, precision and robustness. However, several of these parameters were unchanged between the 2 types of samples studied here (peel *vs*. pulp) and did not need to be repeated. The measurements that did not need to be repeated include the following: (i) the linearity, which concerns the calibration solutions and not the samples themselves; (ii) the accuracy, which was performed as required in a complex matrix devoid of the studied compounds (here, on tomato leaf samples because they do not contain coumarins and furanocoumarins); (iii) the precision; and (iv) the robustness. These latter elements only depended on the equipment. In contrast, the LOD, LOQ, and specificity depended on the matrix containing the chemical compounds and had to be checked again on citrus pulp samples. The LOD, LOQ and specificity were determined using a lime (var. Tahiti, *C*. *aurantifolia (Christm*.*) Swing*.) because it contains both high diversity and quantities of coumarins and furanocoumarins in the pulp.

### Limits of detection and quantitation in citrus pulp

The LOD and LOQ of a lime pulp sample were determined using the same method as Dugrand and colleagues (2013) [39]. Because lime pulp does not contain all 27 of the investigated compounds, this sample was enriched in coumarins and furanocoumarins to confirm the ability to measure the LOD and LOQ for all the studied compounds. The data are presented in S2 Table. The LOD and LOQ values were generally low. Only 6',7'-dihydroxybergamottin, 8-geranyloxypsoralen, byakangelicin and cnidicin had higher limit values. Notably, the LOD and LOQ values in the citrus peel were presented in our previous work [39].

### Specificity

A lime pulp sample was prepared and injected into the UPLC-MS system to check its coumarin and furanocoumarin contents. Based on this analysis, we decided to determine the specificity of 6 quantifiable compounds: aurapten, bergapten, oxypeucedanin, oxypeucedanin hydrate, 8-geranyloxypsoralen and isopimpinellin. For this test, 4 additional lime pulp samples (from the same pulp powder) were spiked with these 6 coumarins and furanocoumarins at 25, 50, 75 and 100% of their initial concentrations. S2 Table shows the equations linking the compounds concentrations, the percentages of the initial concentrations added and the linear correlation coefficients. No matrix effect was observed for the coumarins and furanocoumarins in pulp because the linear correlation coefficients of the equations were above 0.99.

### Statistical analyses

The data were processed using R statistical software (http://www.R-project.org). The gplots R package and the heatmap function were used to build the heatmaps. The qualitative data (0 for the absence of a given compound and 1 for the presence of this compound) were used to create a profile matrix of the 27 coumarins and furanocoumarins detected by UPLC-MS within the 61 *Citrus* varieties. The quantitative data (means of 5 biological replicates expressed as mg.kg^-1^ fresh weight) were used to build (1) Neighbor Joining (NJ) analyses based on the Range Manhattan dissimilarity index (R pvclust package and pvclust function), (2) Principal components analyses (PCAs) where data were transformed as log_10_(1 + x) (R Ade4 package and dudi.pca function) and where accessions of the ancestral taxa were used as active individuals to define the PCA axes and accessions of the secondary species were additional individuals.

## Results

### General coumarin and furanocoumarin diversity in *Citrus*

**Fig 2:**
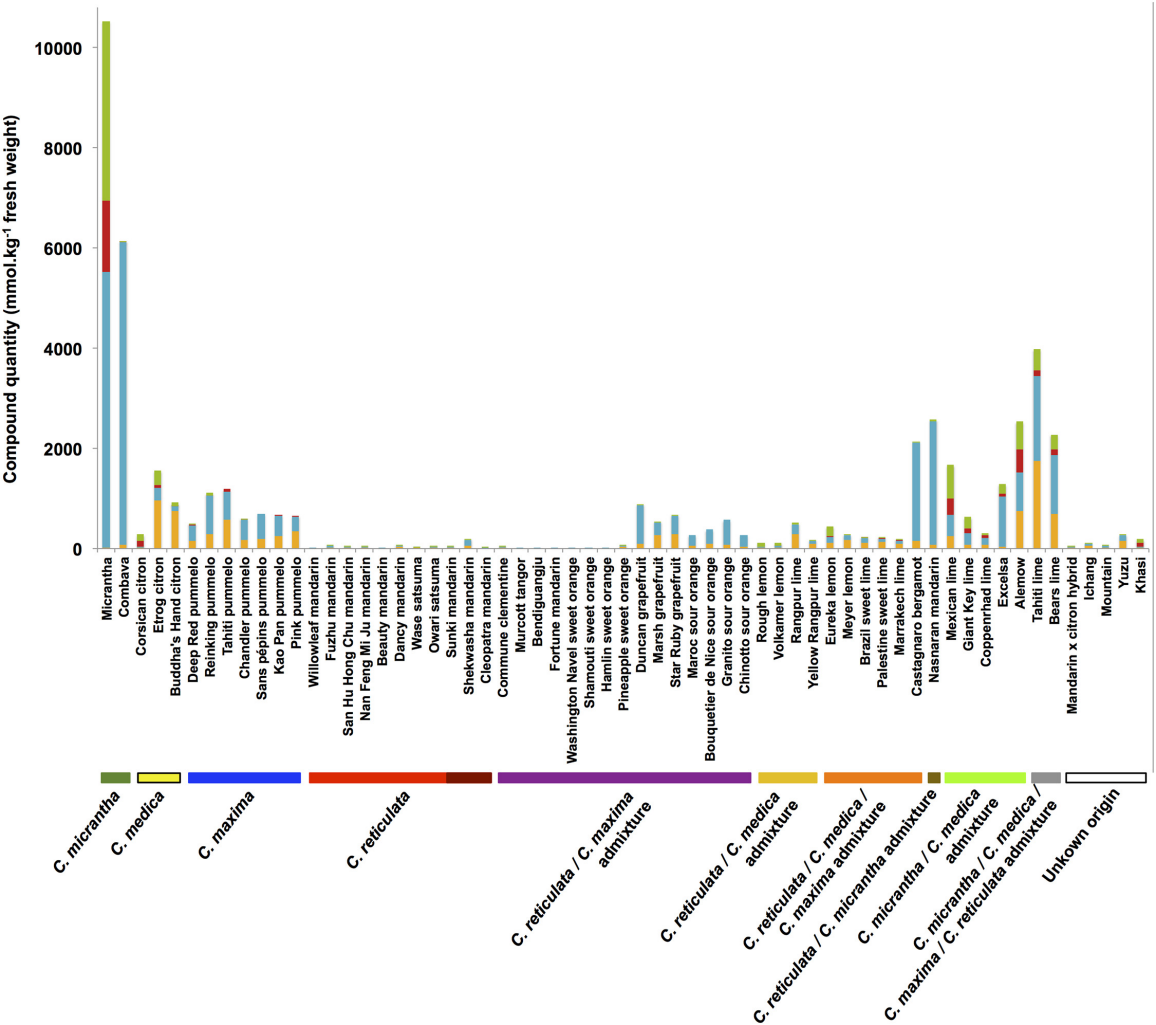
Coumarin and furanocoumarin quantities (mmol kg^-1^ fresh weight) in the peel extracts of the 61 *Citrus* species investigated. Coumarins are represented in orange; furanocoumarins of the bergapten cluster, in blue; furanocoumarins of the xanthotoxin cluster, in red; and furanocoumarins of the isopimpinellin cluster, in green. Ancestral taxa and secondary species are highlighted in the bottom of the graph. Sweet mandarins are presented in bright red, and acidic mandarins are illustrated in deep red.

**Fig 3:**
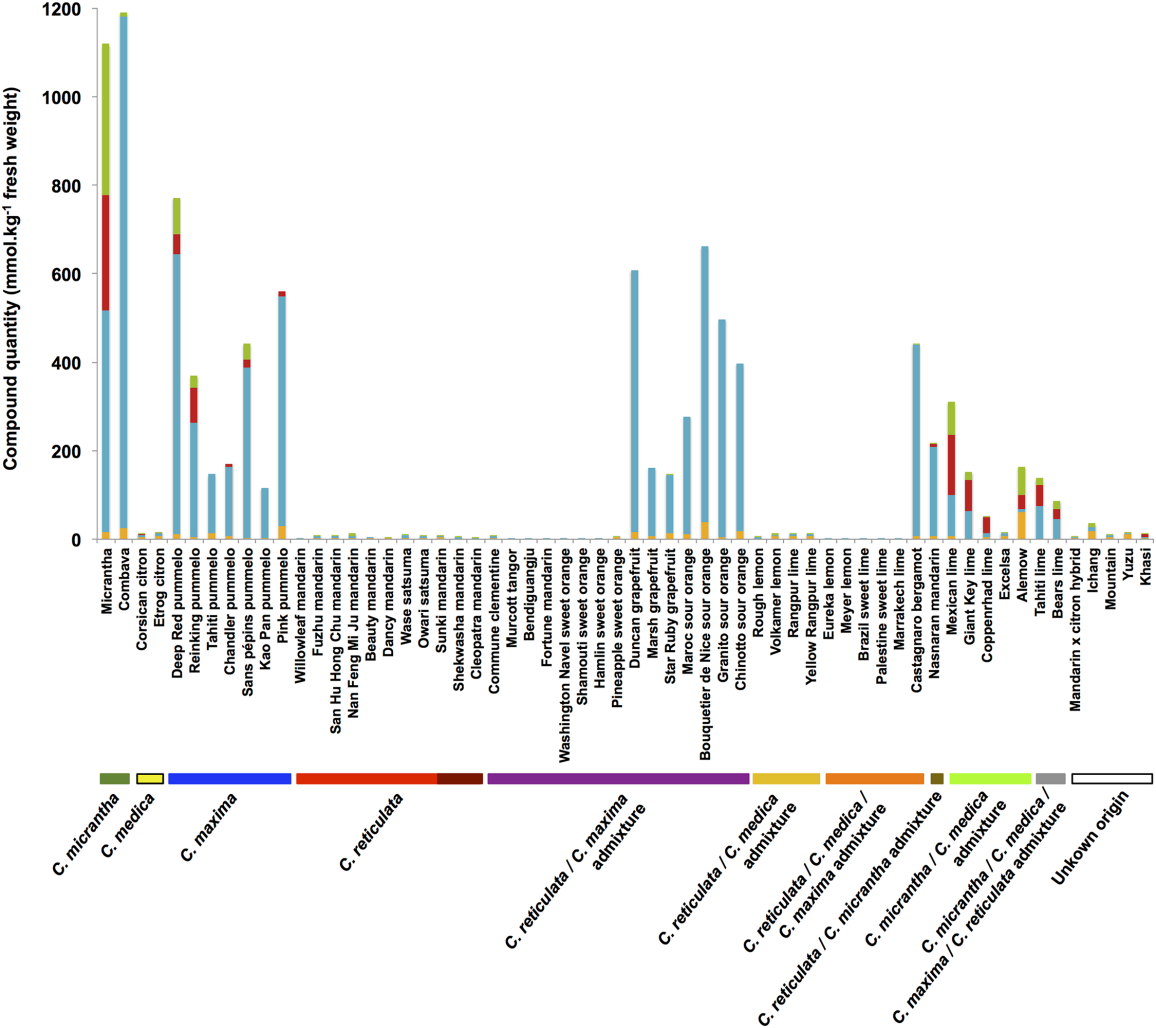
Coumarin and furanocoumarin quantities (mmol kg^-1^ fresh weight) in the pulp extracts of the 61 *Citrus* species investigated. Coumarins are represented in orange; furanocoumarins of the bergapten cluster, in blue; furanocoumarins of the xanthotoxin cluster, in red; and furanocoumarins of the isopimpinellin cluster, in green. Ancestral taxa and secondary species are highlighted in the bottom of the graph. Sweet mandarins are presented in bright red, and acidic mandarins are illustrated in deep red.

In total, 5 coumarins and 21 furanocoumarins were quantitatively determined in the peel and pulp samples of 61 *Citrus* varieties. In addition, 5-geranyloxy-7-methoxycoumarin was unequivocally determined when present in a sample but could not be quantitated due to the insufficient amount of standard available to establish calibration curves. The coumarin and furanocoumarin contents in the citrus peel and pulp samples are presented in Figs 2 and 3, respectively, and are highly variable between the 61 *Citrus* varieties analyzed (see Supporting information, S1 File for complete quantitative results). Generally, citrus fruit peel has broader diversity of compounds than the corresponding pulp, as shown by the total numbers of coumarins and furanocoumarins found in the peel (649 occurrences) and pulp (561 occurrences) samples of the 61 studied *Citrus* varieties. Similar to the diversity of compounds, higher compound concentrations were generally found in peel than in pulp (Figs 2 and 3). Exceptions to this latter rule, when the pulp appears more concentrated than the peel, were observed in the cases of Deep Red and Pink pummelo and Bouquettier de Nice and Chinotto sour oranges for which the pulp is exceptionally rich in furanocoumarins from the bergapten group (Tables A and G in S1 File). Another notable exception was bergamottin, a bergapten-geranylated derivative, which is regularly found more concentrated in the pulp of pummelos (Deep Red, Chandler) and their hybrid grapefruits (Duncan, Marsh) and sour oranges (Maroc, Bouquettier de Nice, Granito) (Tables A, E and G in S1 File). Based on the results presented in Figs 2 and 3, the highest producers of coumarins and furanocoumarins are 2 papedas: Micrantha (10518 and 1118 mmol kg^-1^ fresh weight in peel and pulp, respectively) and Combava (6136 and 1189 mmol kg^-1^ fresh weight in peel and pulp, respectively). Citrons and some of their descendants (particularly Tahiti, Bears, Alemow, Mexican, Excelsa, Giant and Key limes), pummelos and some of their descendants (grapefruit, sour oranges and bergamot) and Nasnaran also synthesize important amounts of coumarins and furanocoumarins with respect to their peel and pulp contents. Other citron descendants (particularly those for which Micrantha is not related to, *e*.*g*., Eureka and Meyer lemons and Marrakech, Palestine, Rangpur and Brazil sweet limes) contain intermediate levels in the peel, compared with extremely low levels in the pulp. Other varieties such as mandarins, small mandarin hybrids, sweet oranges, some limes, some lemons, Ichang papeda and Mountain display low amounts of coumarins and furanocoumarins in both their peel and pulp. As examples, the peel/pulp of Bendiguangju satsuma, Murcott tangor and Shamouti sweet orange present extremely low coumarin and furanocoumarin contents, with 1.85/0.97, 2.10/1.24, and 2.02/1.15 mmol kg^-1^ fresh weight, respectively.

**Fig 4:**
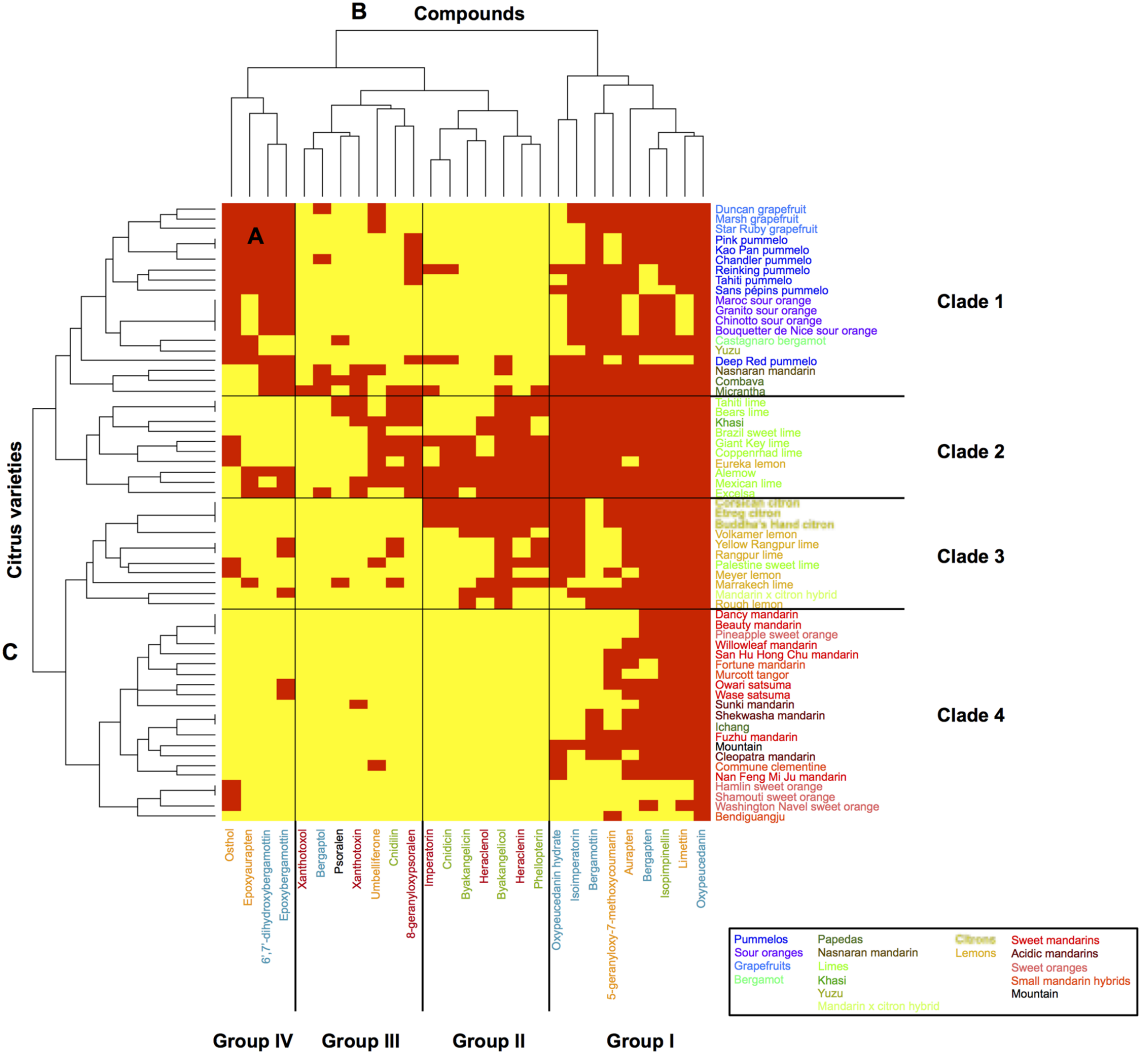
Heatmap displaying the coumarin and furanocoumarin profiles in the peel of the 61 *Citrus* species investigated. A: heatmap; B: dendrogram of compounds; C: dendrogram of *Citrus* varieties. A red square highlights the presence of a given compound, while a yellow square shows its absence. Coumarins are represented in orange; furanocoumarins of the bergapten cluster, in blue; furanocoumarins of the xanthotoxin cluster, in red; and furanocoumarins of the isopimpinellin cluster, in green.

**Fig 5:**
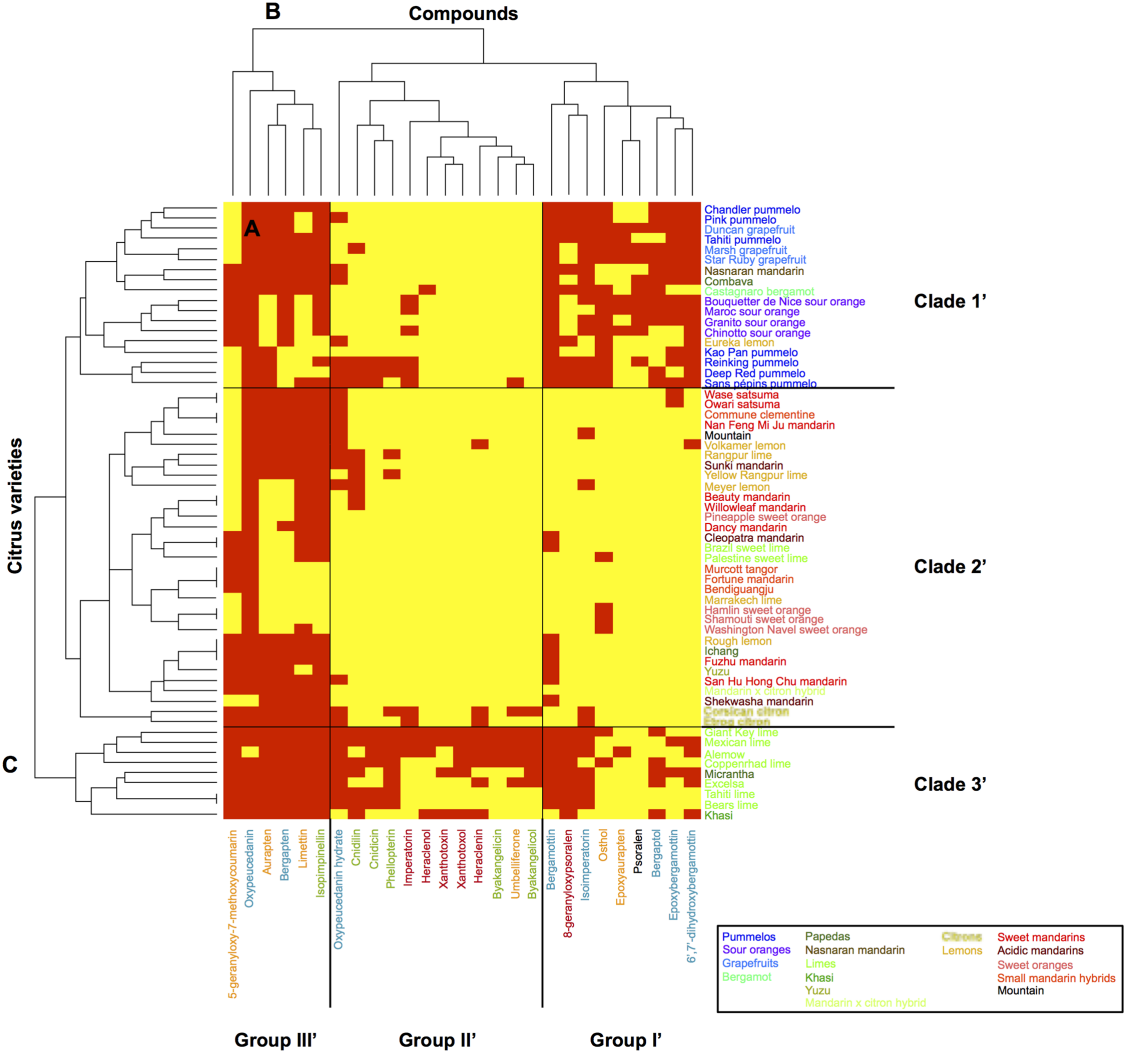
Heatmap displaying the coumarin and furanocoumarin profiles in the pulp of the 61 *Citrus* species investigated. A: heatmap; B: dendrogram of compounds; C: dendrogram of *Citrus* varieties. A red square highlights the presence of a given compound, while a yellow square shows its absence. Coumarins are represented in orange; furanocoumarins of the bergapten cluster, in blue; furanocoumarins of the xanthotoxin cluster, in red; and furanocoumarins of the isopimpinellin cluster, in green.

Heatmap representations associated with hierarchical ascendant classifications (HACs) were chosen to establish classifications and correlations between the chemotypes and *Citrus* varieties. Heatmaps were constructed based on the sole presence or absence (0 for the absence of the compound and 1 for the detection of the compound) of each of the coumarins/furanocoumarins (5-geranyloxy-7-methoxycoumarin included). This analysis allowed the chemotypes observed for the peel (Fig 4) and for the pulp (Fig 5) in all *Citrus* varieties to be summarized.

Based on the largest compound diversity found in peel (heatmap, Fig 4A), coumarins and furanocoumarins were classified by HACs into 4 different subgroups (dendrogram, Fig 4B).

Group I is composed of 3 coumarins (aurapten, limettin, and 5-geranyloxy-7-methoxycoumarin), bergapten and all its dimethylallylated derivatives (isoimperatorin, oxypeucedanin, and oxypeucedanin-hydrate), and the geranylated compounds bergamottin and isopimpinellin. This group includes the most widespread coumarins/furanocoumarins in the *Citrus* species studied, as exemplified by oxypeucedanin, which was ubiquitously found in the peel of all 61 investigated varieties.

Group II is composed of xanthotoxin dimethylallylated compounds (imperatorin, heraclenin, and heraclenol) associated with isopimpinellin dimethylallylated derivatives, where DMAPP is attached to the oxygen bound to C8 (phellopterin, byakangelicol, byakangelicin, and cnidicin).

Group III includes parent compounds such as psoralen, bergaptol, xanthotoxol and umbelliferone, which subsequently generate additional diversified compounds in the coumarin/furanocoumarin pathways. Group III also contains cnidilin, a double DMAPP-derivative of isopimpinellin, and 8-geranyloxypsoralen. Group III includes some of the most scarcely found coumarins/furanocoumarins among the studied *Citrus* varieties.

Group IV is composed of two coumarins, osthol and epoxyaurapten, which are associated with two bergapten geranylated compounds (epoxybergamottin and 6-7-dihydroxybergamottin). Compounds from group IV are less common in *Citrus* varieties and are restricted to some horticultural groups.

The distribution of coumarins and furanocoumarins in citrus pulp (heatmap Fig 5A) significantly differs from that previously described in the peel (heatmap, Fig 4A). In pulp, coumarin/furanocoumarin classifications are only limited to 3 groups (dendrogram, Fig 5B), denoted as I’, II’, and III’ to avoid confusion with the groups described previously in Fig 5.

Group I’ is composed of the coumarins osthol and epoxyaurapten and the furanocoumarins psoralen, the 5 other compounds from the bergapten cluster (prenylated and geranylated forms) and 8-geranyloxypsoralen. Most of the compounds from group I’ are geranylated forms.

Group II’ contains the coumarin umbelliferone, the furanocoumarin oxypeucedanin hydrate, the compounds from the xanthotoxin cluster (except 8-geranyloxypsoralen) and the prenylated compounds of the isopimpinellin cluster. These coumarins and furanocoumarins from group II’ are primarily dimethylallylated compounds.

Group III’ includes compounds that can be found in many *Citrus* fruits: the coumarins 5-geranyloxy-7-methoxycoumarin, aurapten and limettin and the furanocoumarins oxypeucedanin, bergapten and isopimpinellin. Compounds from group III’ are the most widespread in citrus pulp samples.

### Organization of *Citrus* diversity based on coumarin and furanocoumarin contents in peel

Based on the largest compound diversity found in peel (heatmap, Fig 4A), *Citrus* varieties can be classified by HACs into 4 clades (dendrogram, Fig 4C).

Clade 1 gathers *Citrus* from the pummelo group, *i*.*e*., 7 pummelos and pummelo hybrids (3 grapefruits, 4 sour oranges, and 1 bergamot), which are associated with 2 papedas (Combava, Micrantha) and 2 other hybrids: Yuzu and Nasnaran. These varieties primarily synthesize compounds from the groups I and IV, including all the coumarins biosynthetically originating from the parent compound umbelliferone (osthol, epoxyaurapten, aurapten, limettin, 5-geranyloxy, and 7-methoxycoumarin; Fig 1) and furanocoumarins from the bergapten cluster: bergapten, bergaptol and its geranylated derivatives (bergamottin, epoxybergamottin and 6',7'-dihydroxybergamottin), and its dimethylallylated derivatives (isoimperatorin, oxypeucedanin and oxypeucedanin hydrate; Fig 4A).

Clade 2 is characterized by the presence of limes (except Palestine), the Khasi hybrid and the Eureka lemon. Compared to the other *Citrus* groups, clade 2 exhibits the greatest coumarin/furanocoumarin diversity as shown in the heatmap representation (Fig 4A), where a large assortment of prenylated coumarins and furanocoumarins derived from bergapten, xanthotoxin and isopimpinellin parent compounds (all 4 groups represented) is displayed.

Clade 3 is composed of the citron group, *i*.*e*., the 3 citrons, the lemons (except Eureka) and the Palestine sweet lime. This clade also includes the mandarin × citron hybrid. Fruit peels from clade 3 are characterized by the occurrence of prenylated coumarins associated with prenylated furanocoumarins from the bergapten, xanthotoxin and isopimpinellin clusters (Fig 4A). This clade 3 chemotype is quite comparable to that found in clade 2, although coumarins and furanocoumarins from the previously described groups III and IV are less represented in clade 3.

Clade 4 is composed of all the mandarins (sweet and acidic), all the small mandarin hybrids, all the sweet oranges, Ichang papeda and the Mountain hybrid. Clade 4 includes species that are characterized by limited chemotype diversity, primarily restricted to compounds associated previously with group I (prenylated coumarins and furanocoumarins from the bergapten cluster, Fig 4A).

**Fig 6:**
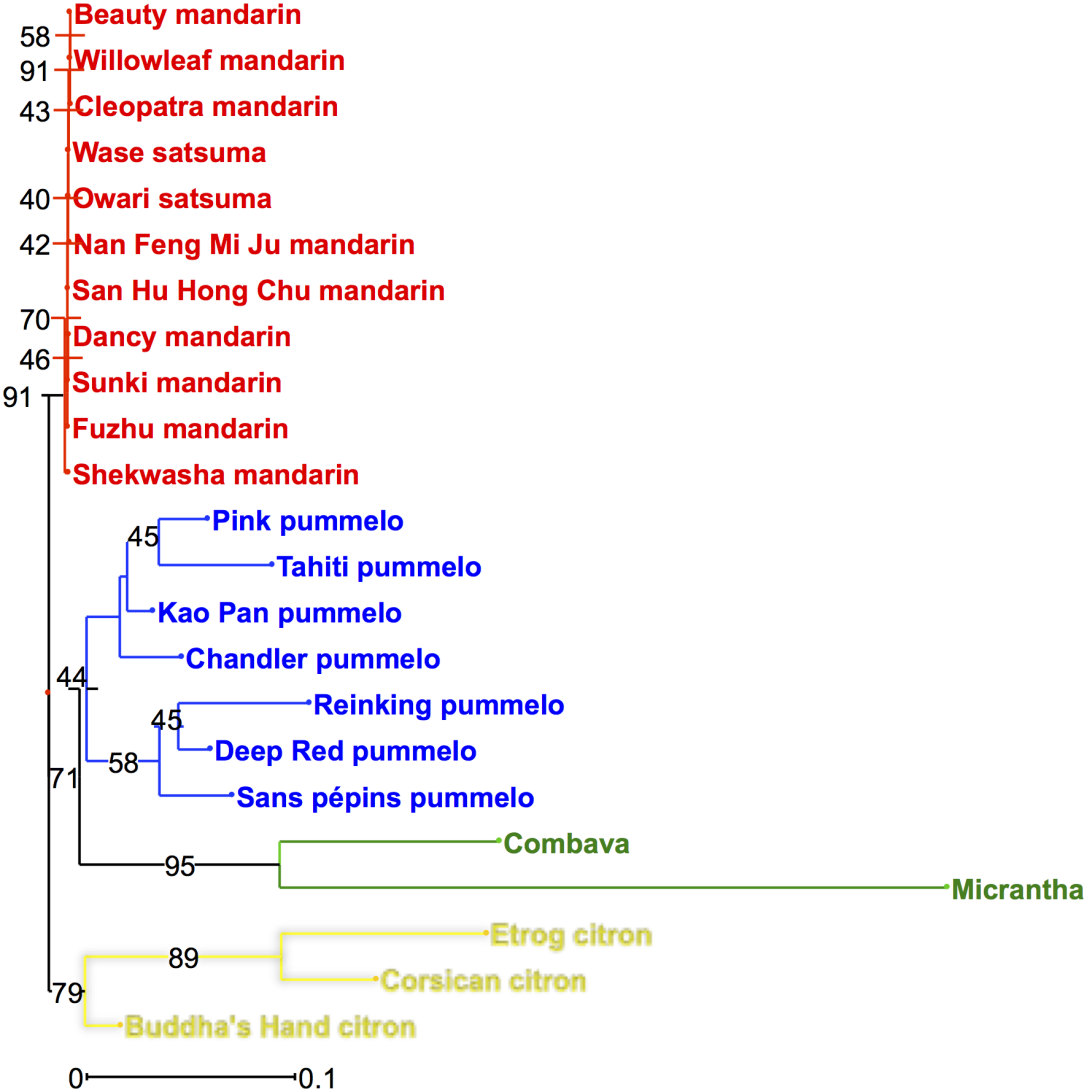
NJ analysis of the 23 citrus varieties belonging to the 4 ancestral taxa based on the coumarins and furanocoumarins contents in peel expressed as mg.kg^-1^ fresh weight. Numbers in black represent the bootstrap probability values. The colors correspond to the phylogenetic constitution of the varieties and are indicated in Figs 2 and 3.

In addition to qualitative analyses, we used the quantitative results of coumarins and furanocoumarins contents in citrus peel to analyze the implication of phylogenetic origins on coumarins and furanocoumarins contents. NJ analyses and PCAs were performed for this purpose. The four ancestral taxa (mandarins, pummelos, papedas and citrons) could be perfectly separated in the NJ analysis based on the quantitative data of coumarins and furanocoumarins in citrus peel (Fig 6). On this NJ analysis, bootstrap values are quite high (91 for mandarins, 71 for pummelos and papedas and 79 for citrons; Fig 6), which demonstrates that it is a strong representation of the structure of citrus diversity. Based on this strong structuration of coumarins and furanocoumarins contents between the four ancestral taxa, we analyzed the coumarins and furanocoumarins contents of the secondary species according to their phylogenomic contents by PCA analysis, using ancestral taxa accessions as active individuals and secondary species accessions as additional individuals.

**Fig 7:**
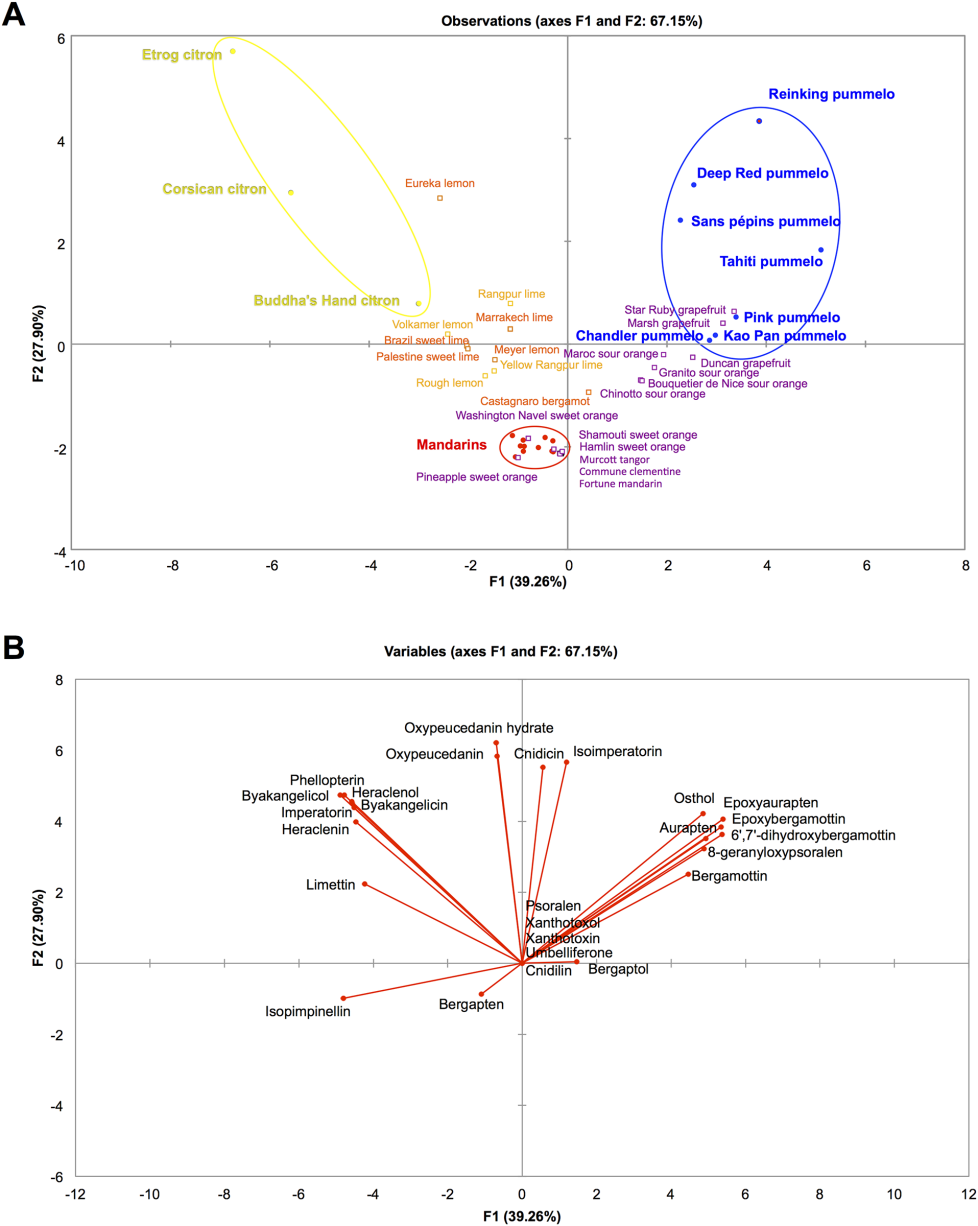
PCA representation of the citrus varieties belonging to the 3 ancestral taxa pummelos, mandarins and citrons and their respective hybrids based on the coumarins and furanocoumarins contents in peel expressed as mg.kg^-1^ fresh weight. A: Distribution of the observations (citrus varieties); B: Distribution of the variables (coumarins and furanocoumarins). Ancestral taxa were used as active individuals (circles) to build the PCA, while secondary species are supplementary individuals (squares). Quantitative data were transformed as log_10_(1 + x). The colors correspond to the phylogenetic constitution of the varieties and are indicated in Figs 2 and 3.

When using the accessions of the four ancestral taxa, the very high coumarins and furanocoumarins contents of papedas (*C*. *micrantha* and *C*. *hystrix*) and some of their hybrids resulted in a very strong impact of these accessions on the PCA axes definition (S1 Fig), even though the quantitative data were transformed as log_10_(1 + x). As a result, the resolution of the differentiation between the three other basic taxa was low and thus the relative position of secondary species difficult to infer (S1 Fig). Therefore, to analyze the organization of coumarins and furanocoumarins contents of the three other ancestral taxa (pummelos, mandarins and citrons) and their respective hybrids, we performed a PCA analysis with only the corresponding accessions (Fig 7). The structure of the diversity of citrus varieties is well represented on the first two axes of this PCA with a value above 67% (Fig 7). Pummelos, mandarins and citrons are well separated on the PCA and the secondary species are well distributed among these ancestral taxa (Fig 7A). For example, varieties with a *C*. *maxima* × *C*. *reticulata* admixture structure are placed on an axis between these 2 taxa. In the same way, the citrus varieties with a *C*. *medica* × *C*. *reticulata* admixture structure genome are distributed between these 2 ancestral taxa. Pummelos, grapefruits and sour oranges are characterized by their peel content in dimethylallylated coumarins (aurapten, epoxyaurapten and osthol) and dimethylallylated furanocoumarins from the bergapten cluster essentially (bergamottin, epoxybergamottin and 6’, 7’- dihydroxybergamottin) (Fig 7B). In contrast, citrons, lemons and limes are characterized by their peel content in limettin and in dimethylallylated furanocoumarins from the xanthotoxin cluster (imperatorin, heraclenin and heraclenol) and from the isopimpinellin cluster (phellopterin, byakangelicol and byakangelicin) (Fig 7B). Mandarins, sweet oranges and mandarin hybrids are poor producers of coumarins and furanocoumarins, even though they produce isopimpinellin and bergapten (Fig 7B).

The PCA with the four basic taxa (S1 Fig) reveals the position of papeda hybrids that are placed between papedas and citrons. The coumarins and furanocoumarins contents of Alemow and Mexican limes place them closer to citrons than papedas, while Nasnaran is closer to papedas than to mandarins (S1 Fig).

### Organization of *Citrus* diversity based on coumarin and furanocoumarin contents in pulp

The identification of coumarins and furanocoumarins in the pulp allowed 3 clades (Fig 5C) to be distinguished instead of the 4 described previously based on the peel content (Fig 4C). For clarity between Figs 5C and 4C, these 3 new clades are nominated 1’, 2’, and 3’.

Clade 1’ is composed of the 7 pummelos, the 4 sour oranges, the 3 grapefruits, the Combava, the Bergamot, the Nasnaran mandarin and the Eureka lemon. Clade 1’ is composed of the same varieties found previously in clade 1 (Fig 4C), with the exception of Yuzu and Micrantha, which are found only in clade 1, and the Eureka lemon, which is restricted to clade 1’. The pulp of these varieties primarily contains prenylated coumarins and prenylated derivatives from the bergapten cluster classified in groups I’ and III’.

Clade 2’ includes all the sweet and acidic mandarins, all the small mandarin hybrids, all the sweet oranges, the lemons (except Eureka), 2 limes (Brazil and Palestine), the 2 citrons for which the pulp compartment could be investigated (NB: due to the absence of pulp in Buddha’s Hand citron fruits, no investigation could be conducted in the pulp of this third citron variety), Ichang, Mountain, Yuzu and the mandarin × citron hybrid. Clade 2’ is composed of varieties classified previously in clades 1 (Yuzu), 2 (Brazil sweet lime), 3 (citrons, lemons and limes) and 4 (mandarins, small mandarin hybrids, sweet oranges, Mountain and Ichang) based on the peel chemotype. Varieties from clade 2’ show the narrowest diversity in coumarins and furanocoumarins in the fruit pulp, primarily restricted to the compounds classified in group III’.

Clade 3’ contains the limes (except Brazil and Palestine), the Micrantha papeda and Khasi. These varieties are associated with clades 1 (Micrantha) and 2 (limes and Khasi) established previously from the peel chemotype. The pulp observed from clade 3’ varieties shows the most diversified chemotypes, with coumarins and furanocoumarins belonging to groups I’, II’, and III’. In particular, dimethylallylated compounds from the xanthotoxin cluster (*i*.*e*., imperatorin, heraclenin, and heraclenol), as well as the parent compound xanthotoxol and its methoxylated derivative xanthotoxin, are exclusively restricted to some of the clade 3’ fruit pulp.

As we did before, the quantitative data of coumarins and furanocoumarins contents in citrus pulp were used to build a NJ analysis between the four ancestral taxa. This NJ analysis displays a weak representation of the structure of citrus diversity, with low bootstrap values for the main branching and incomplete differentiation between the four basic taxa (S2 Fig). Therefore, citrus pulp coumarins and furanocoumarins contents are not strongly related with phylogenetic differentiation. Thus we do not performed analysis to study the relationship of coumarins and furanocoumarins contents on secondary species and their phylogenetic origin.

## Discussion

### Peel presents the richest coumarin/furanocoumarin content

The results obtained from the peel and pulp of the 61 studied varieties clearly demonstrate that the envelope of the fruit is the richest part in terms of both molecular diversity (Fig 4 *vs*. Fig 5) and compound concentration (Figs 2 and 3, Tables A to L in S1 File). Peel prominence regarding coumarin and furanocoumarin contents must be related to the presence of lysogenic cavities in the flavedo (*i*.*e*., pigmented pericarp of the peel), where large quantities of essential oils and polyphenolics are stored. The occurrence of specialized cells involved in the biosynthesis of coumarins and furanocoumarins has been hypothesized in young grapefruits (*C*. *paradisi* ‘Duncan’) [43]: epithelial cells surrounding the secretory cavities show a high level of transcripts potentially committed to the biosynthetic routes, which most likely reflects their specialization for coumarin and furanocoumarin biosynthesis. Future studies aimed at classifying *Citrus* varieties based on coumarin/furanocoumarin chemotypes should be limited to the peel compartment only.

### Coumarin and furanocoumarin diversity found in *Citrus* reflects the organization of the biosynthetic pathways

Qualitative studies are useful to understand the organization of the biosynthetic pathways, as they reflect the ability of varieties to produce different metabolites. HACs based on the presence/absence of coumarins/furanocoumarins in peel allowed 4 categories of compounds to be distinguished (Fig 4B, groups I to IV). Each group is logically structured with coumarins and furanocoumarins engaged in the same cascade of enzymatic reactions (*i*.*e*., groups of substrates and products either leading to the same reaction end product or originating from the same precursor compound).

Group I is composed of one dimethylallylated coumarin (aurapten), one geranylated coumarin (5-geranyloxy, 7-methoxycoumarin) and one dimethoxy coumarin (limettin). All these products originate from the biosynthetic transformation of the parent compound umbelliferone. In accordance with the rule that substrates and products from the same biosynthetic route are regrouped, group I is composed of the 3 prenylated derivatives that form after the dimethylallylation of bergaptol (*i*.*e*., isoimperatorin, oxypeucedanin, and oxypeucedanin hydrate) and one prenylated derivative (bergamottin) that forms after the geranylation of the same bergaptol.

Notably, group I has the C5, C8 dimethoxylated furanocoumarin isopimpinellin and the C5 mono-methoxylated bergapten, both of which are ubiquitously found in *Citrus*, whereas xanthotoxin, the C8-isomer of bergapten that occurs in group III, is primarily restricted to *Citrus* from clade 2 (limes and papedas). At present, the route leading from psoralen to isopimpinellin is not established, and whether isopimpinellin is formed after C5 methoxylation of xanthotoxin, C8 methoxylation of bergapten, or after both is unknown [16,35]. Based on our data regarding the strong co-occurrence of isopimpinellin and bergapten, isopimpinellin is likely derived from bergapten in *Citrus*.

Interestingly, the 3 compounds that form after *O*-geranylation of bergaptol (bergamottin → epoxybergamottin → 6’, 7’, dihydroxybergamottin) are not clustered in the same group: bergamottin (*i*.*e*., 5 geranyloxy-psoralen), the first compound, is located in group I, and the latter 2 epoxy derivatives are in group IV. Symmetrically, group IV contains epoxyaurapten, another epoxy derivative for which the parent geranyloxy compound (aurapten) is located in group I. Because compounds from group I are represented in almost all *Citrus* accessions and because compounds from group IV are principally limited to pummelos and their hybrids, it is tempting to hypothesize that the enzymes responsible for the synthesis of *O*-geranylated compounds (*i*.*e*., aromatic *O*-prenyltransferases) might be ubiquitously spread in *Citrus*, whereas the enzymes in charge of the synthesis of the two epoxydes (epoxybergamottin and epoxyaurapten) and of 6’,7’,dihydroxybergamottin might be more scarcely distributed and restricted to the pummelo group (clade 1). Group IV also includes osthol, the unique *C*-prenylated compound investigated in this study. Notably, the distribution of this *C*-prenylated structure is much more limited than other *O*-prenylated coumarins (aurapten, 5-geranyloxy, and 7-methoxycoumarin), although it is difficult to draw general conclusions regarding the distribution *C vs*. *O* prenyltransferases due to the lack of *C*-prenylated compounds analyzed in this study.

Group II is exclusively composed of *O*-prenylated compounds characterized by a dimethylallyloxy chain bound at C8 of the furanocoumarin core structure. These compounds include imperatorin, heraclenin, and heraclenol from the xanthotoxin cluster and cnidicin, phellopterin, byakangelicol, and byakangelicin from the isopimpinellin cluster. They are principally found in *Citrus* clade 2 (limes and papedas) and clade 3 (citrons, lemons, and sour limes). Whether a single aromatic *O*-prenyltransferase might operate on multiple substrates such as xanthotoxol and 8-hydroxybergapten or whether different enzymes are engaged in the synthesis of imperatorin and phellopterin remain unknown. This point is presently difficult to address because the number of aromatic prenyltransferases described from plants thus far is limited to only seven. Notably, five of these seven aromatic prenyltransferases appear to be extremely specific regarding the prenyl donors that they accept (DMAPP, GPP) [44–46,38], whereas 2 prenyltransferases described recently from hop plant show affinity for both DMAPP and GPP [47]. Four of these enzymes also demonstrate a high specificity for prenyl acceptors, as they only tolerate a single aromatic entity as a substrate [44–46,38], including a *C*-prenyltransferase described recently from lemon that is involved in the synthesis of 8-geranylumbelliferone [46]. A 5^th^ enzyme isolated from *Sophora flavescens* accepts more promiscuous aromatic substrates, as it is able to prenylate 2 structurally close isoflavones: genistein and its methylated derivative biochanin A [45]. Consistent with this *Sophora flavescens* enzyme, the two hop prenyltransferases described recently show affinity for different substrates such as acylphloroglucinols and naringenin chalcone [47].

Group III primarily contains intermediate compounds that are subsequently transformed into prenylated/methoxylated/epoxydated derivatives such as psoralen, xanthotoxol, bergaptol, xanthotoxin, and umbelliferone. These intermediate compounds are the most scarcely found among citrus fruit peel (Fig 4A) because they are rapidly converted into more chemically diverse end products. Group III also includes cnidilin and 8-geranyloxypsoralen, two prenylated compounds rarely found in *Citrus*.

### Coumarin and furanocoumarin chemical diversity in relation to the recent advances in *Citrus* phylogeny

Based on molecular markers and sequencing, 4 ancestral taxa, *C*. *maxima*, *C*. *medica*, *C*. *reticulata* and *C*. *micrantha*, have been identified in *Citrus* [8,10,12,14]. These basic taxa also could be well distinguished based on the coumarins and furanocoumarins contents in their peel (Fig 6). Ancestral taxa present no evidence of interspecific introgression, except in sweet mandarin, which presents approximately 10% introgression of *C*. *maxima* [13,14]. However, the acidic mandarin ‘Cleopatra’, ‘Sunki’ and ‘Shekwasha’ present no evidence of introgression [14]. The coumarin and furanocoumarin chemotypes observed in fruit peel and pulp were compared within the 4 ancestral taxa and were relatively homogeneous inside the taxa, except in papedas. *C*. *maxima* presents relatively high coumarin and furanocoumarin contents characterized by the presence of all coumarins and bergapten-related compounds (bergapten, bergaptol and its geranylated derivatives, bergamottin, epoxybergamottin, and 6',7'-dihydroxybergamottin) (Figs 2 and 3, Table A in S1 File). *C*. *reticulata* presents the lowest coumarin and furanocoumarin contents (Figs 2 and 3, Table D in S1 File). This taxon is characterized by the total absence of xanthotoxin in peel and pulp. No difference was observed between sweet and acidic mandarin. *C*. *medica* is characterized by the occurrence of prenylated coumarins associated with prenylated furanocoumarins from bergapten, xanthotoxin and isopimpinellin clusters (Figs 2 and 3, Table B in S1 File). The Papeda taxon is not cultivated, except Combava, and remains the least known of all ancestral taxa. Only few genetic resources for this group are available in the different germplasm banks in the world, and extreme profiles can be observed within this group. Micrantha and Combava present the highest furanocoumarin content (from bergapten, xanthotoxin and isopimpinellin) (Figs 2 and 3, Table C in S1 File). However, we must keep in mind that Micrantha was harvested outside Corsica, where all other samples were collected, and that different pedo-climatic conditions might influence coumarin and furanocoumarin synthesis [48]. In contrast, Ichang papeda, also considered a true papeda, has very low coumarin and furanocoumarin contents (Figs 2 and 3, Table C in S1 File). In different studies [8,15], *C*. *ichangensis* (Ichang papeda) is grouped with the wild mandarins *C*. *tachibana* and *C*. *mangshanensis*. Unfortunately, these wild mandarins were unavailable to our study because these genotypes are only present in Asia. However, these species are considered genetically distinct from other mandarins although they clustered with them. Our results are concordant with these studies, as *C*. *Ichangensis* is localized with all the mandarin accessions.

All other species result from hybridization between these four ancestral taxa. The coumarins and furanocoumarins found in these secondary species display contrasting molecular diversity associated with variable quantitative traits. Many of these species, such as *C*. *sinensis*, *C*. *aurantium*, *C*. *paradisi*, and tangors, originate from *C*. *reticulata* x *C*. *maxima* hybridization, with variable contributions of each taxon. For the tangors, the contribution of *C*. *reticulata* is higher (from 63% to 89%) [49]. *C*. *sinensis* presents a contribution of 60% of *C*. *reticulata* and 40% of *C*. *maxima*, *C*. *aurantium* 50% and 50%; and *C*. *paradisi* approximately 40% and 60% respectively [49]. Mandarin hybrids (tangors) and sweet orange, closely related to mandarins, are logically present in the coumarin/furanocoumarin mandarin clade (clade IV, Fig 4C). In the same way, these secondary species are closer to the mandarin cluster on the PCA based on the citrus peel content in coumarins and furanocoumarins (Fig 7A). This taxonomical group is of special interest because it contains the lowest coumarin and furanocoumarin producers amongst all the studied *Citrus* species. In contrast, *C*. *aurantium* and *C*. *paradisi* have similar coumarin and furanocoumarin profiles compared with *C*. *maxima* (Fig 4C, clade I and Fig 7A). All these results show a strong concordance between phytochemical and molecular data.

*C*. *limon* (Swingle and Reece taxonomy) is more complex and originates from 2 or 3 taxa hybridization. *C*. *limon* can result from a direct hybridization between *C*. *medica* and *C*. *reticulata* (‘Volkamer lemon’, ‘Rough lemon’ and ‘Rangpur’ lime) or more complex hybridization between 3 ancestral taxa, *C*. *medica*, *C*. *reticulata*, and *C*. *maxima* (‘Meyer’, ‘Eureka’ lemons and Marrakech lime) [49]. Most of these species are present in the same clade 3 as citron (*C*. *medica*, Fig 4C) or are placed on an axis between the citron and mandarin clusters on the PCA based on the quantitative results in citrus peel (Fig 7A).

Similarly, *C*. *aurantifolia* originates from different taxa hybridization (2 to 4 taxa) composed systematically of citron and papeda taxons. ‘Alemow’, ‘Excelsa’, Mexican’ and Giant key limes display extremely similar phylogenomic origins, with close to 50% contributions from *C*. *medica* and *C*. *micrantha* (14). *C*. *aurantifolia* ‘Palestine’ and ‘Brazil’ sweet lime have phylogenetic structures similar to *C*. *limon* (‘Meyer’, ‘Eureka’ lemons, and ‘Marrakech’ lime). *C*. *aurantifolia* ‘Palestine’ and ‘Brazil’ sweet lime contains close to 50% *C*. *medica* and displayed 2 other ancestral taxa, *C*. *reticulata* and *C*. *maxima*, with a greater contribution from *C*. *reticulata* (32–34%) than *C*. *maxima* (15–20% in ‘Palestinian’ sweet lime) [14,49]. Coppenrhad lime is a triploid originating from a (*C*. *micrantha x C*. *medica)* x *C*. *medica* hybridization with a diploid gamete of the *C*. *micrantha x C*. *medica* parent [49]. Tahiti and Bearss lime, which are also triploid, have contributions from the 4 ancestral taxa (1/6 *C*. *maxima*, 1/3 *C*. *medica*, 1/3 *C*. *micrantha* and 1/6 *C*. *reticulata*) [49]. All of these secondary species containing Papeda, except Palestine sweet lime, are grouped in the same clade 2 Fig 4C), indicating that our phytochemical data are consistent with the recent molecular data cited previously. The mandarin × citron hybrid is a simple hybrid of *C*. *medica x C*. *reticulata* [49] as Rough lemon, Rangpur lime and Volkamer lemon. This hybrid has similar profiles as the coumarin and furanocoumarin citron profiles and is grouped in clade 3 (Fig 4C).

Bergamot is considered to have emerged from a lemon X sour orange crossing and contains 40% of pummelo [49], which is consistent with its position in the pummelo clade. In the same way, bergamot is placed between pummelos and mandarins, close to the sour oranges, on the PCA based on the quantitative results of coumarins and furanocoumarins in peel (Fig 7A). With reference to our survey, this finding could be due to the persistence of some chemical compounds that might have been inherited from pummelo such as osthol, epoxyaurapten and bergamottin (Figs 4 and 7).

The origin of the Mountain hybrid remains undetermined; however, this hybrid has the same maternal origin as papeda [10]. Yuzu might be derived from the hybridization between Ichang papeda and a mandarin [5]. These 2 hybrids are present in the mandarin clade, which also contains the Ichang papeda displaying extremely low coumarin and furanocoumarin contents. Thus, based on our phytochemical data, these hybrids are most likely derived from Ichang papeda and/or mandarin.

The Khasi hybrid displays coumarin and furanocoumarin profiles similar to Micrantha papeda, allowing us to draw the hypothesis of affiliation with this cultivar.

Nasnaran mandarin is a direct mandarin-papeda hybrid [14] with a papeda mitotype [10]. This hybrid presents a strong papeda fragrance, although it looks similar to a mandarin. Here, its profile is more closely related to papedas, particularly Combava, which might suggest a parental link between them.

In brief, all secondary species resulting from a cross directly involving the ancestral taxa *C*. *maxima* (sour oranges and grapefruits), *C*. *reticulata* (sweet oranges and some tangors), *C*. *medica* (some lemons) or *C*. *micrantha* (some limes and Nasnaran mandarin) present coumarin and furanocoumarin profiles similar to the parent ancestral taxa, with a prevalence of the chemotypes found in pummelos, citrons and papedas over mandarins and, identically, a prevalence of the chemotypes found in papedas over citrons. These findings constitute valuable information for future breeding programs that will allow the coumarin and furanocoumarin contents in *Citrus* species to be reduced.

### Breeding programs for selection against coumarins and furanocoumarins in *Citrus*: Global and targeted approaches

We observed previously that the lowest coumarin and furanocoumarin contents are found in sweet and acidic mandarins and most of their hybrids (including sweet oranges and small mandarin hybrids), Ichang papeda and the 2 hybrids Yuzu and Mountain. In contrast, pummelos, citrons and papedas, which contain the highest quantities of coumarins and furanocoumarins, generate hybrids that are themselves rich in these compounds. This finding led us to hypothesize that breeding programs aimed at decreasing the toxic coumarin and furanocoumarin contents in *Citrus* should preferentially involve mandarins or Ichang papeda. *C*. *sinensis* is a good example of the possibility of obtaining a low coumarin and furanocoumarin hybrid from *C*. *reticulata*, although it has a relatively high genetic background from pummelo (40%) [14]. This hypothesis is in agreement with a study evaluating the inhibition of the CYP3A4 by immature *Citrus* fruit extracts [50]. In this work, *C*. *paradisi* was shown to be the most effective inhibitor of CYP3A4, while *C*. *unshiu* displayed relatively weak inhibition of the cytochrome most likely due to low furanocoumarin content. Other *Citrus* fruits were tested; their capacities to inhibit CYP3A4 are ordered as follows: *C*. *paradisi* > *C*. *limon* > *C*. *aurantium* > *C*. *grandis* (also called *C*. *maxima*) > *C*. *reticulata* > *C*. *sinensis* > *C*. *unshiu* [50]. This order is globally consistent with our quantitative results, considering that the coumarin and furanocoumarin contents vary greatly from one species to another and depend on environmental conditions.

**Fig 8:**
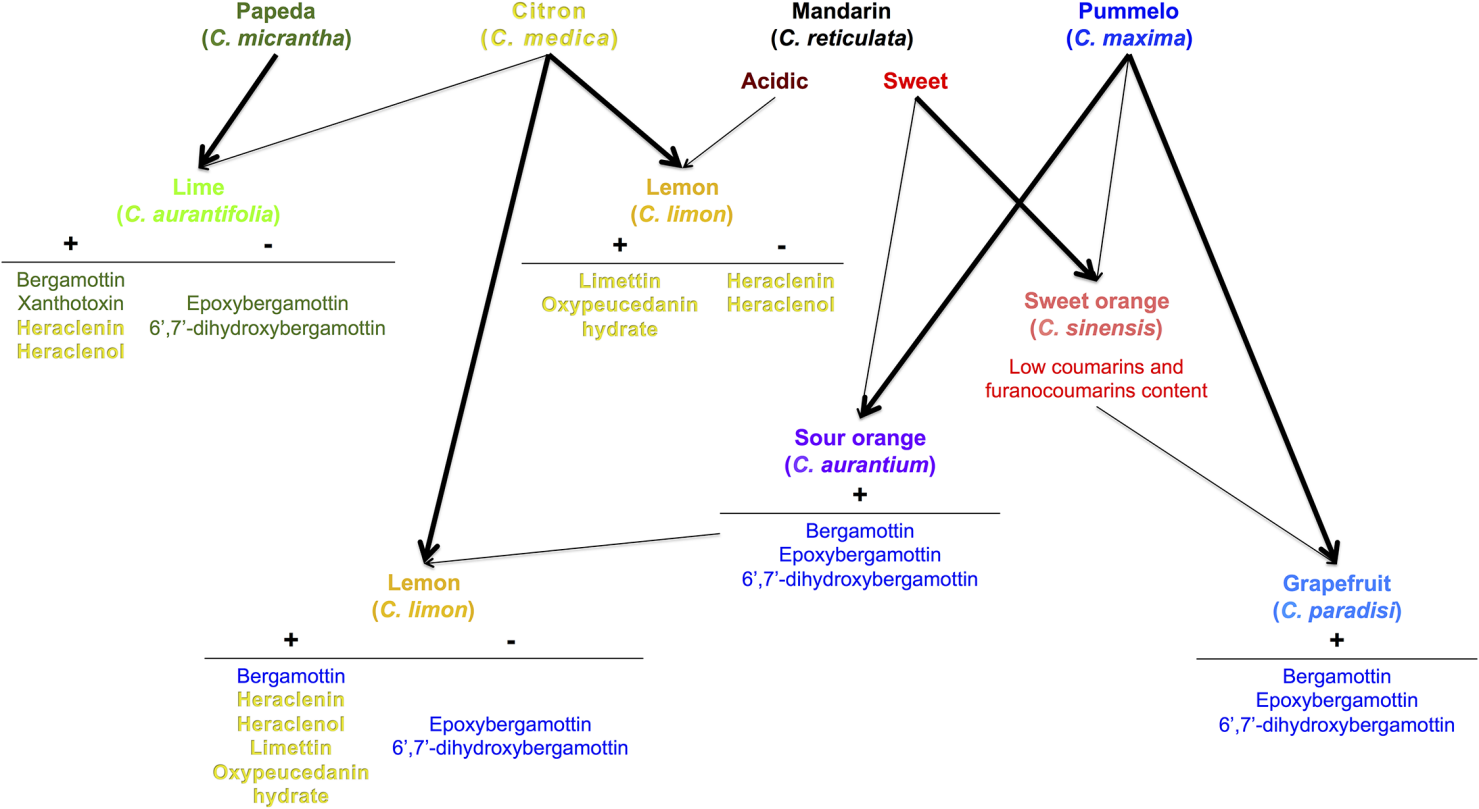
Schematic representation of coumarin/furanocoumarin inheritance from the 4 ancestral taxa (pummelo, mandarin, citron, and papeda) in the cultivated *Citrus* species. Parental relations between species are illustrated by arrows. Thick arrows represent high chemotype similarities between hybrids and their ascendants. Same colors were used as in Figs 4 and 5 for representing *Citrus* species. Coumarins and furanocoumarins are indicated in the same color as the ancestral taxon that transmitted them to their descendants. The symbol “+” indicates the probable transmission of compounds in the secondary species, while the symbol “-”indicates their disappearance.

Pummelos are high producers of geranylated compounds from the bergapten cluster: bergamottin, epoxybergamottin and 6',7'-dihydroxybergamottin. These chemical compounds are phototoxic and are strong inhibitors of CYP3A4 [20,51,52]. Sour oranges and grapefruits are direct descendants of pummelos that also produce these 3 compounds, and their other parents (sweet mandarin and sweet orange, respectively) generally do not produce them. Therefore, we can hypothesize that crosses involving pummelos and mandarins or sweet oranges usually include the toxic furanocoumarins bergamottin, epoxybergamottin and 6',7'-dihydroxybergamottin (Fig 8). This is in accordance with a recent statement issued by Xu and coworkers, who confirmed that pummelos as parents have a definite contribution to the furanocoumarins content (bergapten, bergamottin, epoxybergamottin and 6’,7’-dihydroxybergamottin) of grapefruits [42]. However, sweet oranges and small mandarin hybrids that have a greater contribution of *C*. *reticulata* in their genotype have lost the capacity to produce these 3 compounds (Fig 8). This finding suggests that the genes encoding the enzyme machinery involved in the biosynthesis of bergamottin, epoxybergamottin and 6',7'-dihydroxybergamottin in pummelos might be dominant and transmitted to their direct hybrid progenies. This hypothesis of dominant genes is consistent with molecular data regarding coumarin/furanocoumarin biosynthesis available to date, where the capacity to synthesize such compounds is given by very specific enzymes encoded by specialized genes such as dioxygenases, prenyltransferases, and cytochrome P450s [35–38,46].

Crosses involving citrons are interesting, with the objective to primarily remove the toxic epoxybergamottin and 6',7'-dihydroxybergamottin introduced by pummelos (see lemon progenies: Eureka, Meyer and Marrakech) or by *C*. *micrantha* (see lime progenies) (Fig 8). However, bergamottin and the phototoxic xanthotoxin [53] from Micrantha are maintained in some limes, and citrons bring other phototoxic compounds to their descendants such as heraclenin and heraclenol [51,54], which are absent in pummelos and Micrantha (Fig 8). Acidic mandarins might be a solution to prevent the transmission of heraclenin and heraclenol from citrons because among the 4 *C*. *reticulata* × *C*. *medica* lemon hybrids investigated (Rough, Rangpur, Yellow Rangpur and Volkamer), only the Volkamer lemon produces these 2 compounds (Fig 8). Alternatively, citrons can also bring the phototoxic limettin and oxypeucedanin hydrate [51] to lemons (see Eureka, Meyer and Marrakech lemons progenies), 2 chemical compounds absent in sour oranges (Fig 8). This problem cannot be solved by the use of mandarins as the parental line (see Rough, Rangpur and Volkamer lemons progenies) because they all contain limettin and sometimes oxypeucedanin hydrate (Fig 8).

In conclusion, this study highlights the different chemotypes found in *Citrus* species regarding coumarin and furanocoumarin diversity and content. Three of 4 ancestral taxa (pummelos, citrons and papedas) synthesize high amounts of these compounds, whereas mandarins, from the last ancestral taxon, appear practically devoid of them. As for hybrid species, their corresponding chemotypes appear inherited from the 4 ancestral taxa, with strong hypotheses of a prevalence of pummelos, citrons and papedas over mandarins and of papedas over citrons. Compared to other species, mandarins are of invaluable interest for *Citrus* breeding programs aimed at removing toxic furanocoumarins in *Citrus*. Particularly, sweet oranges and small mandarins hybrids, which have a higher contribution of mandarin in their genotype, do not produce the toxic compounds bergamottin, epoxybergamottin and 6',7'-dihydroxybergamottin. An alternative strategy for the future would lie in the identification of an upstream gene involved in the first steps leading to the furanocoumarin pathway. Such gene characterization should allow gene editing strategies to be performed and would constitute an efficient solution for removing these toxic compounds from *Citrus* species.

## Supporting Information

S1 Fig PCA representation of the 61 citrus varieties based on the coumarins and furanocoumarins contents in peel expressed as mg.kg^-1^ fresh weight. Ancestral taxa were used as active individuals (circles) to build the PCA, while secondary species are supplementary individuals (squares). Quantitative data were transformed as log_10_(1 + x). The colors correspond to the phylogenetic constitution of the varieties and are indicated in Figs 2 and 3.
(TIF)

S2 Fig NJ analysis of the 23 citrus varieties belonging to the 4 ancestral taxa based on the coumarins and furanocoumarins contents in pulp expressed as mg.kg^-1^ fresh weight. Numbers in black represent the bootstrap probability values. The colors correspond to the phylogenetic constitution of the varieties and are indicated in Figs 2 and 3.
(TIF)

S1 File Concentrations (in mg kg^-1^ fresh weight ± standard deviation) of the coumarins and the furanocoumarins in the peel and pulp extracts of the 61 examined citrus species. (PDF)

S1 Table List of the 61 citrus species investigated in this study and their phylogenetic constitutions. (PDF)

S2 Table Limits of detection and quantitation of the coumarins and furanocoumarins in the citrus pulp and specificity (equation and coefficient of determination, r^2^) of the UPLC-MS method. (PDF)
