# Supplementary figures and images for: The Distribution of Coumarins and Furanocoumarins in Citrus Species Closely Matches Citrus Phylogeny and Reflects the Organization of Biosynthetic Pathways
Source: PLoS One. 2015 Nov 11;10(11):e0142757. doi: 10.1371/journal.pone.0142757 (PMC4641707; doi:10.1371/journal.pone.0142757)

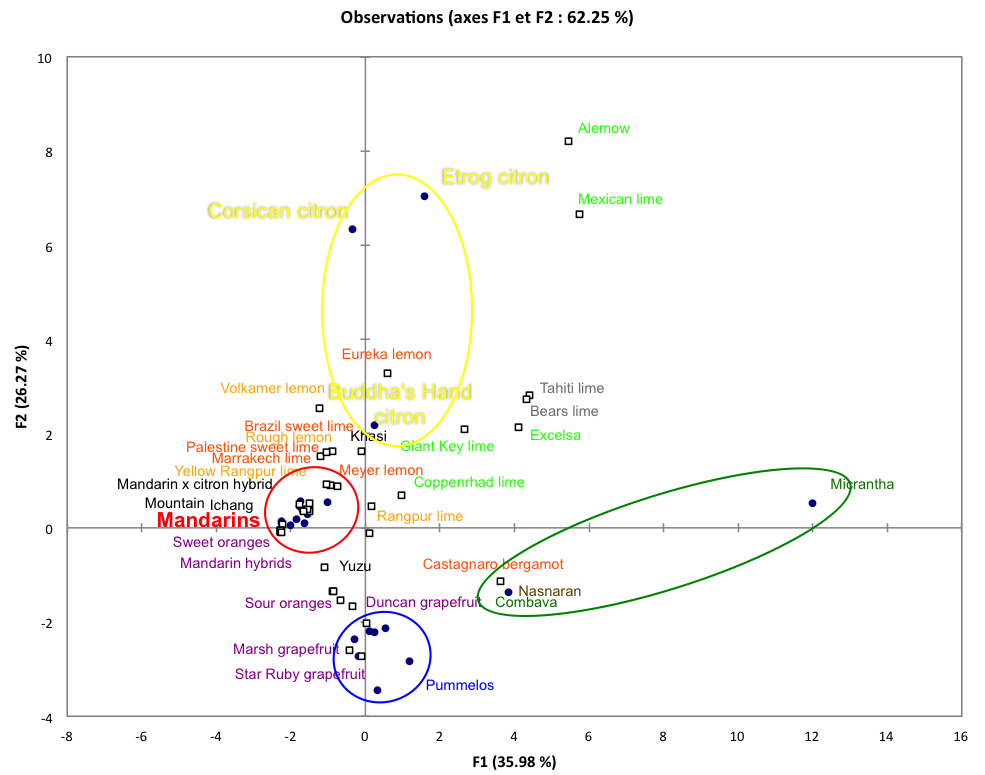

Supplement: S1 Fig — Ancestral taxa were used as active individuals (circles) to build the PCA, while secondary species are supplementary individuals (squares). Quantitative data were transformed as log10(1 + x). The colors correspond to the phylogenetic constitution of the varieties and are indicated in Figs 2 and 3. (TIF) [file pone.0142757.s001.tif]

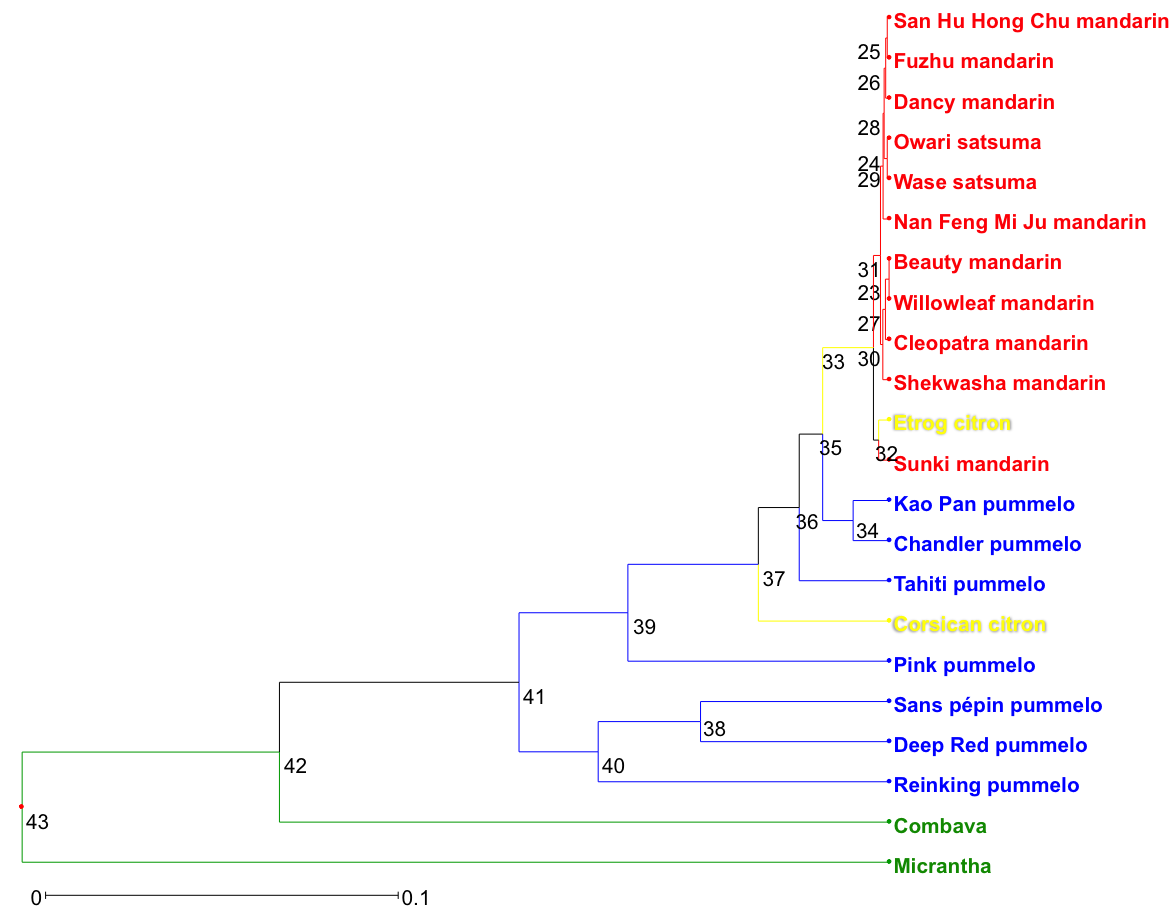

Supplement: S2 Fig — Numbers in black represent the bootstrap probability values. The colors correspond to the phylogenetic constitution of the varieties and are indicated in Figs 2 and 3. (TIF) [file pone.0142757.s002.tif]
