# Supplementary material for: The Distribution of Coumarins and Furanocoumarins in Citrus Species Closely Matches Citrus Phylogeny and Reflects the Organization of Biosynthetic Pathways
Source: PLoS One. 2015 Nov 11;10(11):e0142757. doi: 10.1371/journal.pone.0142757 (PMC4641707; doi:10.1371/journal.pone.0142757)
Supplement: S1 File — (PDF) [file pone.0142757.s003.pdf]

| | | Deep Red pummelo | | Reinking pummelo | | Tahiti pummelo | | Chandler pummelo | |
|---|---|---|---|---|---|---|---|---|---|
| | | Peel | Pulp | Peel | Pulp | Peel | Pulp | Peel | Pulp |
| | **Coumarins** | | | | | | | | |
| | Umbelliferone | | | | | | | | |
| | Osthol | 16.66 ± 3.83 | 0.18 ± 0.08 | 8.92 ± 3.27 | Traces | 22.18 ± 9.81 | Traces | 1.46 ± 0.39 | Traces |
| | Aurapten | 7.15 ± 0.47 | 2.98 ± 1.31 | 15.17 ± 7.62 | 1.46 ± 0.68 | 16.87 ± 4.29 | Traces | 39.34 ± 15.12 | 2.12 ± 0.88 |
| | Epoxyaurapten | 21.99 ± 2.50 | | 60.34 ± 8.17 | | 133.19 ± 25.75 | Traces | 10.45 ± 1.46 | |
| | Limettin | | | 2.86 ± 0.45 | | 2.56 ± 0.79 | 2.79 ± 0.86 | 1.96 ± 0.12 | Traces |
| | 5-geranyloxy-7-methoxycoumarin | | | Detected | | Detected | | | |
| | **Furanocoumarins** | | | | | | | | |
| | Psoralen | | | | Traces | | | | |
| Bergapten cluster | Bergaptol | | Traces | | | | | 13.83 ± 2.11 | Traces |
| | Bergapten | | | | | | Traces | 26.80 ± 7.28 | Traces |
| | Isoimperatorin | 27.95 ± 8.41 | 132.66 ± 40.93 | 74.66 ± 33.80 | 26.26 ± 8.22 | Traces | Traces | | Traces |
| | Oxypeucedanin | 31.22 ± 10.29 | 4.96 ± 2.58 | 34.12 ± 5.54 | 17.02 ± 8.95 | 0.68 ± 0.16 | 0.77 ± 0.28 | 0.90 ± 0.12 | 0.59 ± 0.07 |
| | Oxypeucedanin hydrate | 9.98 ± 0.67 | 21.74 ± 7.52 | 9.51 ± 2.77 | 25.67 ± 3.63 | | | | |
| | Bergamottin | 0.24 ± 0.05 | 0.46 ± 0.19 | 3.24 ± 1.03 | Traces | 14.66 ± 6.76 | 2.62 ± 1.83 | 1.48 ± 0.75 | 12.33 ± 5.67 |
| | Epoxybergamottin | 9.31 ± 3.00 | | 82.41 ± 37.55 | Traces | 77.31 ± 20.18 | 21.03 ± 9.58 | 10.76 ± 1.19 | 5.28 ± 1.06 |
| | 6',7'-dihydroxybergamottin | 13.36 ± 3.07 | 19.45 ± 7.01 | 34.55 ± 10.84 | 6.59 ± 2.18 | 108.56 ± 40.20 | 24.42 ± 12.07 | 60.27 ± 4.52 | 38.14 ± 6.77 |
| Xanthotoxin cluster | Xanthotoxol | | | | | | | | |
| | Xanthotoxin | | | | | | | | |
| | Imperatorin | Traces | 10.38 ± 1.08 | Traces | 21.33 ± 1.48 | | | | |
| | Heraclenin | | | | | | | | |
| | Heraclenol | | | | | | | | |
| | 8-geranyloxypsoralen | 1.47 ± 0.05 | 2.43 ± 0.75 | 4.02 ± 0.95 | Traces | 15.29 ± 8.18 | Traces | 2.59 ± 0.63 | 2.52 ± 0.96 |
| Isopimpinellin cluster | Isopimpinellin | | | Traces | Traces | Traces | Traces | 0.54 ± 0.05 | Traces |
| | Phellopterin | | 3.26 ± 0.67 | | Traces | | | | |
| | Byakangelicol | 0.65 ± 0.19 | | | | | | | |
| | Byakangelicin | | | | | | | | |
| | Cnidilin | | 0.71 ± 0.15 | | Traces | | | | |
| | Cnidicin | 9.20 ± 2.14 | 24.46 ± 5.30 | 14.17 ± 1.94 | 9.63 ± 4.25 | | | | |
| | TOTAL (mg.kg$^{-1}$) | 149.18 | 223.67 | 343.97 | 107.96 | 391.30 | 51.63 | 170.38 | 60.98 |

| | | Sans pépin pummelo | | Kao Pan pummelo | | Pink pummelo | |
|---|---|---|---|---|---|---|---|
| | | Peel | Pulp | Peel | Pulp | Peel | Pulp |
| | **Coumarins** | | | | | | |
| | Umbelliferone | | Traces | | | | |
| | Osthol | 27.84 ± 8.70 | 0.19 ± 0.06 | 1.69 ± 0.63 | Traces | 20.69 ± 6.25 | Traces |
| | Aurapten | 11.28 ± 4.91 | 0.36 ± 0.12 | 33.46 ± 9.80 | 0.69 ± 0.16 | 41.92 ± 16.85 | 9.09 ± 4.04 |
| | Epoxyaurapten | 11.99 ± 2.77 | | 39.78 ± 9.29 | | 32.20 ± 14.87 | |
| | Limettin | 1.68 ± 0.48 | Traces | 1.62 ± 0.30 | | 1.96 ± 0.57 | |
| | 5-geranyloxy-7-methoxycoumarin | Detected | | | | | |
| | **Furanocoumarins** | | | | | | |
| | Psoralen | | | | | | |
| Bergapten cluster | Bergaptol | | 2.38 ± 0.68 | | | | Traces |
| | Bergapten | | | 20.58 ± 3.58 | | 22.91 ± 2.58 | 12.09 ± 2.19 |
| | Isoimperatorin | 67.61 ± 13.52 | 88.39 ± 51.26 | | | | Traces |
| | Oxypeucedanin | 37.38 ± 6.62 | 5.08 ± 1.42 | Traces | 0.33 ± 0.07 | Traces | 0.60 ± 0.13 |
| | Oxypeucedanin hydrate | 11.68 ± 5.08 | 4.50 ± 1.71 | | | | 1.46 ± 0.65 |
| | Bergamottin | 0.20 ± 0.09 | 0.09 ± 0.04 | 0.58 ± 0.20 | 6.77 ± 2.64 | 2.55 ± 0.88 | 17.89 ± 8.11 |
| | Epoxybergamottin | 11.30 ± 5.80 | Traces | 51.57 ± 17.58 | Traces | 17.35 ± 2.85 | Traces |
| | 6',7'-dihydroxybergamottin | 18.34 ± 8.02 | 5.61 ± 2.05 | 56.03 ± 22.27 | 34.41 ± 12.23 | 50.40 ± 8.55 | 149.97 ± 68.00 |
| Xanthotoxin cluster | Xanthotoxol | | | | | | |
| | Xanthotoxin | | | | | | |
| | Imperatorin | | 4.71 ± 2.09 | | | | |
| | Heraclenin | | | | | | |
| | Heraclenol | | | | | | |
| | 8-geranyloxypsoralen | | Traces | 2.40 ± 0.64 | | 1.75 ± 0.29 | 3.52 ± 0.70 |
| Isopimpinellin cluster | Isopimpinellin | | Traces | Traces | | Traces | Traces |
| | Phellopterin | | | | | | |
| | Byakangelicol | | | | | | |
| | Byakangelicin | | | | | | |
| | Cnidilin | | Traces | | | | |
| | Cnidicin | | 12.53 ± 2.41 | | | | |
| | TOTAL (mg.kg$^{-1}$) | 199.30 | 123.84 | 207.71 | 42.20 | 191.73 | 194.62 |

**Tables A. Concentration (in mg.kg-1 fresh weight ± standard deviation) of the coumarins and the furanocoumarins in the pummelos peel and pulp extracts.** The mention « Traces » is used for compounds that could be detected but not quantitated (3 < S/N < 10) while « Detected » is used when 5-geranyloxy-7-methoxycoumarin was identified in the samples.

| | | Corsican citron | | Etrog citron | | Buddha's Hand citron | |
|---|---|---|---|---|---|---|---|
| | | Peel | Pulp | Peel | Pulp | Peel | Pulp |
| | **Coumarins** | | | | | | |
| | Umbelliferone | | 0.20 ± 0.01 | | | | |
| | Osthol | | | | | | |
| | Aurapten | 0.56 ± 0.08 | 0.40 ± 0.15 | 0.49 ± 0.11 | 0.36 ± 0.07 | 0.68 ± 0.21 | |
| | Epoxyaurapten | | | | | | |
| | Limettin | 3.92 ± 0.54 | 0.66 ± 0.09 | 198.95 ± 20.55 | 1.33 ± 0.09 | 152.25 ± 24.24 | |
| | 5-geranyloxy-7-methoxycoumarin | Detected | Detected | Detected | Detected | Detected | |
| | **Furanocoumarins** | | | | | | |
| | Psoralen | | | | | | |
| Bergapten cluster | Bergaptol | | | | | | |
| | Bergapten | 2.57 ± 0.44 | 0.35 ± 0.02 | 8.83 ± 2.30 | 0.85 ± 0.18 | 6.54 ± 0.83 | |
| | Isoimperatorin | 0.96 ± 0.09 | Traces | 4.44 ± 0.87 | Traces | 3.09 ± 0.81 | |
| | Oxypeucedanin | 1.32 ± 0.22 | 0.42 ± 0.02 | 40.84 ± 9.30 | 0.19 ± 0.00 | 13.83 ± 3.67 | |
| | Oxypeucedanin hydrate | 0.48 ± 0.08 | 0.21 ± 0.05 | 16.47 ± 3.72 | 0.16 ± 0.01 | 6.18 ± 0.64 | |
| | Bergamottin | | | | | | |
| | Epoxybergamottin | | | | | | |
| | 6',7'-dihydroxybergamottin | | | | | | |
| Xanthotoxin cluster | Xanthotoxol | | | | | | |
| | Xanthotoxin | | | | | | |
| | Imperatorin | 3.95 ± 0.96 | 0.26 ± 0.06 | 2.98 ± 0.89 | 0.21 ± 0.02 | Traces | |
| | Heraclenin | 18.79 ± 2.68 | 0.21 ± 0.05 | 4.15 ± 1.57 | Traces | 0.66 ± 0.15 | |
| | Heraclenol | 7.23 ± 1.40 | | 7.44 ± 1.20 | | Traces | |
| | 8-geranyloxypsoralen | | | | | | |
| Isopimpinellin cluster | Isopimpinellin | 1.78 ± 0.16 | 0.33 ± 0.03 | 5.30 ± 0.50 | 0.68 ± 0.05 | 6.66 ± 0.51 | |
| | Phellopterin | 5.10 ± 0.75 | Traces | 15.91 ± 2.67 | | 1.94 ± 0.15 | |
| | Byakangelicol | 13.33 ± 2.85 | 0.06 ± 0.01 | 34.86 ± 9.17 | | 10.11 ± 1.21 | |
| | Byakangelicin | 22.40 ± 5.92 | | 29.38 ± 12.05 | | Traces | |
| | Cnidilin | | | | | | |
| | Cnidicin | Traces | | 5.10 ± 1.22 | | Traces | |
| | TOTAL (mg.kg$^{-1}$) | 82.39 | 3.10 | 375.14 | 3.78 | 201.94 | |

**Table B. Concentration (in mg.kg-1 fresh weight ± standard deviation) of the coumarins and the furanocoumarins in the citrons peel and pulp extracts.** The mention « Traces » is used for compounds that could be detected but not quantitated (3 < S/N < 10) while « Detected » is used when 5-geranyloxy-7-methoxycoumarin was identified in the samples. No data could be obtained for the pulp of Buddha’s hand citron as the fruits of this citrus species did not contain pulp.

| | | Micrantha | | Combava | | Ichang papeda | |
|---|---|---|---|---|---|---|---|
| | | Peel | Pulp | Peel | Pulp | Peel | Pulp |
| | **Coumarins** | | | | | | |
| | Umbelliferone | | | | | | |
| | Osthol | | | | | | |
| | Aurapten | 4.03 ± 1.43 | 1.66 ± 0.49 | 1.00 ± 0.09 | 0.52 ± 0.10 | 1.45 ± 0.65 | 1.57 ± 0.85 |
| | Epoxyaurapten | | | | | | |
| | Limettin | 2.93 ± 0.42 | 2.27 ± 0.43 | 14.39 ± 3.52 | 4.92 ± 0.43 | 11.16 ± 2.99 | 2.65 ± 0.38 |
| | 5-geranyloxy-7-methoxycoumarin | Detected | Detected | Detected | Detected | | Detected |
| | **Furanocoumarins** | | | | | | |
| | Psoralen | | | Traces | 0.19 ± 0.02 | | |
| Bergapten cluster | Bergaptol | 21.54 ± 10.22 | 13.31 ± 3.06 | 25.85 ± 8.59 | 57.98 ± 2.64 | | |
| | Bergapten | 728.32 ± 165.98 | 32.41 ± 8.66 | 17.28 ± 4.12 | 4.86 ± 0.64 | 7.58 ± 1.81 | 1.53 ± 0.53 |
| | Isoimperatorin | 21.61 ± 3.43 | 1.46 ± 0.41 | 18.36 ± 3.61 | 3.65 ± 0.53 | | |
| | Oxypeucedanin | 274.97 ± 93.72 | 6.19 ± 1.87 | 895.14 ± 65.03 | 16.97 ± 1.39 | Traces | 0.31 ± 0.07 |
| | Oxypeucedanin hydrate | 71.52 ± 11.68 | 19.40 ± 7.38 | 107.49 ± 14.01 | 95.12 ± 14.69 | | |
| | Bergamottin | 47.23 ± 10.57 | 28.49 ± 11.65 | 23.07 ± 3.19 | 29.40 ± 1.92 | Traces | 0.28 ± 0.11 |
| | Epoxybergamottin | 108.42 ± 44.67 | 12.43 ± 3.11 | 485.91 ± 23.72 | 53.70 ± 7.92 | | |
| | 6',7'-dihydroxybergamottin | 111.39 ± 19.60 | 28.08 ± 10.60 | 314.38 ± 51.12 | 82.63 ± 19.44 | | |
| Xanthotoxin cluster | Xanthotoxol | 4.37 ± 1.52 | 3.90 ± 1.46 | | | | |
| | Xanthotoxin | 32.26 ± 5.26 | 6.87 ± 2.18 | 0.47 ± 0.15 | | | |
| | Imperatorin | Traces | | | | | |
| | Heraclenin | | | | | | |
| | Heraclenol | | | | | | |
| | 8-geranyloxypsoralen | 420.99 ± 195.40 | 70.78 ± 22.45 | | | | |
| Isopimpinellin cluster | Isopimpinellin | 694.07 ± 298.76 | 65.63 ± 19.96 | 5.33 ± 1.03 | 2.12 ± 0.47 | 5.09 ± 0.49 | 2.09 ± 0.70 |
| | Phellopterin | 13.51 ± 2.69 | 3.90 ± 0.37 | | | | |
| | Byakangelicol | 107.27 ± 37.92 | 5.56 ± 0.95 | | | | |
| | Byakangelicin | | | | | | |
| | Cnidilin | 113.40 ± 16.07 | 13.10 ± 4.23 | | | | |
| | Cnidicin | | | | | | |
| | TOTAL (mg.kg$^{-1}$) | 2777.83 | 315.44 | 1908.67 | 352.06 | 25.28 | 8.43 |

**Table C. Concentration (in mg.kg-1 fresh weight ± standard deviation) of the coumarins and the furanocoumarins in the papedas peel and pulp extracts.** The mention « Traces » is used for compounds that could be detected but not quantitated (3 < S/N < 10) while « Detected » is used when 5-geranyloxy-7-methoxycoumarin was identified in the samples.

| | | Willowleaf mandarin | | Fuzhu mandarin | | San Hu Hong Chu mandarin | | Nan Feng Mi Ju mandarin | |
|---|---|---|---|---|---|---|---|---|---|
| | | Peel | Pulp | Peel | Pulp | Peel | Pulp | Peel | Pulp |
| | **Coumarins** | | | | | | | | |
| | Umbelliferone | | | | | | | | |
| | Osthol | | | | | | | | |
| | Aurapten | Traces | | 0.31 ± 0.11 | 0.11 ± 0.02 | 0.30 ± 0.07 | 0.14 ± 0.04 | 1.41 ± 0.24 | 0.29 ± 0.07 |
| | Epoxyaurapten | | | | | | | | |
| | Limettin | 1.18 ± 0.28 | 0.25 ± 0.06 | 5.44 ± 1.00 | 0.59 ± 0.19 | 3.68 ± 1.70 | 0.52 ± 0.13 | 3.17 ± 0.47 | 0.35 ± 0.02 |
| | 5-geranyloxy-7-methoxycoumarin | | | Detected | Detected | Detected | Detected | | |
| | **Furanocoumarins** | | | | | | | | |
| | Psoralen | | | | | | | | |
| Bergapten cluster | Bergaptol | | | | | | | | |
| | Bergapten | Traces | | 4.72 ± 0.77 | 0.81 ± 0.06 | 3.74 ± 1.82 | 0.49 ± 0.07 | 2.71 ± 0.57 | 0.30 ± 0.06 |
| | Isoimperatorin | | | | | | | | |
| | Oxypeucedanin | 0.36 ± 0.05 | 0.22 ± 0.05 | 1.95 ± 0.44 | 0.20 ± 0.04 | 1.42 ± 0.64 | 0.15 ± 0.01 | 1.15 ± 0.06 | 0.22 ± 0.02 |
| | Oxypeucedanin hydrate | | | | | | 0.32 ± 0.05 | Traces | 0.38 ± 0.06 |
| | Bergamottin | | | 0.36 ± 0.14 | 0.06 ± 0.03 | | 0.07 ± 0.03 | | |
| | Epoxybergamottin | | | | | | | | |
| | 6',7'-dihydroxybergamottin | | | | | | | | |
| Xanthotoxin cluster | Xanthotoxol | | | | | | | | |
| | Xanthotoxin | | | | | | | | |
| | Imperatorin | | | | | | | | |
| | Heraclenin | | | | | | | | |
| | Heraclenol | | | | | | | | |
| | 8-geranyloxypsoralen | | | | | | | | |
| Isopimpinellin cluster | Isopimpinellin | Traces | Traces | 2.69 ± 0.32 | 0.55 ± 0.18 | 2.11 ± 0.95 | 0.33 ± 0.03 | 5.02 ± 0.42 | 1.88 ± 0.36 |
| | Phellopterin | | | | | | | | |
| | Byakangelicol | | | | | | | | |
| | Byakangelicin | | | | | | | | |
| | Cnidilin | | Traces | | | | | | |
| | Cnidicin | | | | | | | | |
| | TOTAL (mg.kg$^{-1}$) | 1.54 | 0.47 | 15.47 | 2.32 | 11.25 | 2.02 | 13.46 | 3.42 |

| | | Beauty mandarin | | Dancy mandarin | | Wase Satsuma | | Owari Satsuma | |
|---|---|---|---|---|---|---|---|---|---|
| | | Peel | Pulp | Peel | Pulp | Peel | Pulp | Peel | Pulp |
| | **Coumarins** | | | | | | | | |
| | Umbelliferone | | | | | | | | |
| | Osthol | | | | | | | | |
| | Aurapten | | | | | 0.63 ± 0.12 | 0.26 ± 0.05 | 0.64 ± 0.07 | 0.15 ± 0.05 |
| | Epoxyaurapten | | | | | | | | |
| | Limettin | 1.53 ± 0.47 | 0.27 ± 0.13 | 7.97 ± 0.52 | 0.53 ± 0.12 | 2.11 ± 0.25 | 0.43 ± 0.08 | 2.93 ± 0.47 | 0.43 ± 0.15 |
| | 5-geranyloxy-7-methoxycoumarin | | | | | | | Detected | |
| | **Furanocoumarins** | | | | | | | | |
| | Psoralen | | | | | | | | |
| Bergapten cluster | Bergaptol | | | | | | | | |
| | Bergapten | Traces | | 4.85 ± 0.40 | Traces | 2.12 ± 0.30 | 0.56± 0.06 | 2.54 ± 0.17 | Traces |
| | Isoimperatorin | | | | | | | | |
| | Oxypeucedanin | 0.35 ± 0.09 | 0.34 ± 0.10 | Traces | Traces | 0.88 ± 0.13 | 0.25 ± 0.04 | 1.06 ± 0.05 | 0.28 ± 0.04 |
| | Oxypeucedanin hydrate | | | | | | Traces | | Traces |
| | Bergamottin | | | | | | | | |
| | Epoxybergamottin | | | | | Traces | 0.80 ± 0.09 | 2.73 ± 0.52 | 1.10 ± 0.23 |
| | 6',7'-dihydroxybergamottin | | | | | | | | |
| Xanthotoxin cluster | Xanthotoxol | | | | | | | | |
| | Xanthotoxin | | | | | | | | |
| | Imperatorin | | | | | | | | |
| | Heraclenin | | | | | | | | |
| | Heraclenol | | | | | | | | |
| | 8-geranyloxypsoralen | | | | | | | | |
| Isopimpinellin cluster | Isopimpinellin | Traces | Traces | 2.37 ± 0.13 | 0.27 ± 0.04 | 1.10 ± 0.14 | 0.36 ± 0.03 | 1.41 ± 0.08 | 0.34 ± 0.09 |
| | Phellopterin | | | | | | | | |
| | Byakangelicol | | | | | | | | |
| | Byakangelicin | | | | | | | | |
| | Cnidilin | | Traces | | | | | | |
| | Cnidicin | | | | | | | | |
| | TOTAL (mg.kg$^{-1}$) | 1.88 | 0.61 | 15.19 | 0.80 | 6.84 | 2.66 | 11.31 | 2.30 |

| | | Sunki mandarin | | Shekwasha mandarin | | Cleopatra mandarin | |
|---|---|---|---|---|---|---|---|
| | | Peel | Pulp | Peel | Pulp | Peel | Pulp |
| | **Coumarins** | | | | | | |
| | Umbelliferone | | | | | | |
| | Osthol | | | | | | |
| | Aurapten | | Traces | 0.73 ± 0.37 | 0.18 ± 0.05 | | |
| | Epoxyaurapten | | | | | | |
| | Limettin | 5.30 ± 0.20 | 0.86 ± 0.17 | 11.23 ± 5.70 | Traces | 0.55 ± 0.17 | Traces |
| | 5-geranyloxy-7-methoxycoumarin | | | | | Detected | Detected |
| | **Furanocoumarins** | | | | | | |
| | Psoralen | | | | | | |
| Bergapten cluster | Bergaptol | | | | | | |
| | Bergapten | 3.86 ± 0.60 | 0.68 ± 0.05 | 23.00 ± 11.51 | 0.80 ± 0.08 | 0.50 ± 0.14 | |
| | Isoimperatorin | | | | | | |
| | Oxypeucedanin | 0.61 ± 0.15 | 0.13 ± 0.01 | 2.18 ± 1.33 | | 0.79 ± 0.18 | 0.33 ± 0.04 |
| | Oxypeucedanin hydrate | | Traces | | | 0.73 ± 0.31 | |
| | Bergamottin | | | 0.98 ± 0.52 | Traces | 0.16 ± 0.04 | 0.14 ± 0.03 |
| | Epoxybergamottin | | | | | | |
| | 6',7'-dihydroxybergamottin | | | | | | |
| Xanthotoxin cluster | Xanthotoxol | | | | | | |
| | Xanthotoxin | Traces | | | | | |
| | Imperatorin | | | | | | |
| | Heraclenin | | | | | | |
| | Heraclenol | | | | | | |
| | 8-geranyloxypsoralen | | | | | | |
| Isopimpinellin cluster | Isopimpinellin | 1.52 ± 0.22 | 0.51 ± 0.03 | 5.73 ± 3.15 | 0.66 ± 0.11 | 0.23 ± 0.08 | 0.11 ± 0.01 |
| | Phellopterin | | | | | | |
| | Byakangelicol | | | | | | |
| | Byakangelicin | | | | | | |
| | Cnidilin | | Traces | | | | |
| | Cnidicin | | | | | | |
| | TOTAL (mg.kg$^{-1}$) | 11.29 | 2.18 | 43.85 | 1.64 | 2.96 | 0.58 |

**Tables D. Concentration (in mg.kg-1 fresh weight ± standard deviation) of the coumarins and the furanocoumarins in the sweet and acidic mandarins peel and pulp extracts.** The mention « Traces » is used for compounds that could be detected but not quantitated (3 < S/N < 10) while « Detected » is used when 5-geranyloxy-7-methoxycoumarin was identified in the samples.

| | | Duncan grapefruit | | Marsh grapefruit | | Star Ruby grapefruit | |
|---|---|---|---|---|---|---|---|
| | | Peel | Pulp | Peel | Pulp | Peel | Pulp |
| | **Coumarins** | | | | | | |
| | Umbelliferone | 2.79 ± 0.50 | | 3.38 ± 0.85 | | 2.90 ± 1.16 | |
| | Osthol | 3.24 ± 0.47 | Traces | 3.94 ± 1.25 | 0.09 ± 0.02 | 5.13 ± 2.03 | 0.12 ± 0.04 |
| | Aurapten | 10.89 ± 6.37 | 2.28 ± 0.31 | 36.34 ± 15.93 | 1.66 ± 0.77 | 38.51 ± 25.07 | 3.80 ± 1.54 |
| | Epoxyaurapten | 4.69 ± 0.82 | 2.95 ± 0.39 | 35.96 ± 9.53 | Traces | 32.58 ± 6.71 | Traces |
| | Limettin | 1.23 ± 0.07 | | 1.08 ± 0.30 | Traces | 1.71 ± 0.68 | 0.32 ± 0.09 |
| | 5-geranyloxy-7-methoxycoumarin | Detected | | Detected | | Detected | |
| | **Furanocoumarins** | | | | | | |
| | Psoralen | | 0.36 ± 0.04 | | 0.20 ± 0.11 | | 0.27 ± 0.12 |
| Bergapten cluster | Bergaptol | 17.73 ± 3.93 | 8.26 ± 2.27 | | 3.91 ± 1.19 | | 3.74 ± 0.99 |
| | Bergapten | 3.10 ± 0.65 | Traces | 1.13 ± 0.41 | Traces | 1.54 ± 0.66 | 1.19 ± 0.22 |
| | Isoimperatorin | Traces | Traces | Traces | Traces | | Traces |
| | Oxypeucedanin | 0.71 ± 0.08 | 0.44 ± 0.04 | 0.76 ± 0.33 | 0.29 ± 0.10 | 0.62 ± 0.24 | 0.35 ± 0.04 |
| | Oxypeucedanin hydrate | | | | | | |
| | Bergamottin | 1.87 ± 1.11 | 26.53 ± 5.40 | 6.55 ± 1.99 | 10.46 ± 2.62 | 12.67 ± 4.03 | 13.70 ± 4.81 |
| | Epoxybergamottin | 4.94 ± 1.16 | Traces | 25.57 ± 9.09 | 4.46 ± 1.66 | 51.55 ± 21.46 | 3.43 ± 1.22 |
| | 6',7'-dihydroxybergamottin | 245.64 ± 51.95 | 175.13 ± 5.50 | 55.94 ± 15.80 | 34.21 ± 6.44 | 68.97 ± 26.28 | 20.82 ± 7.90 |
| Xanthotoxin cluster | Xanthotoxol | | | | | | |
| | Xanthotoxin | | | | | | |
| | Imperatorin | | | | | | |
| | Heraclenin | | | | | | |
| | Heraclenol | | | | | | |
| | 8-geranyloxypsoralen | | Traces | | | | |
| Isopimpinellin cluster | Isopimpinellin | 0.47 ± 0.05 | Traces | 0.38 ± 0.08 | Traces | 0.95 ± 0.21 | 0.14 ± 0.04 |
| | Phellopterin | | | | | | |
| | Byakangelicol | | | | | | |
| | Byakangelicin | | | | | | |
| | Cnidilin | | | | Traces | | |
| | Cnidicin | | | | | | |
| | TOTAL (mg.kg$^{-1}$) | 297.30 | 215.95 | 171.03 | 55.28 | 217.13 | 47.88 |

**Table E. Concentration (in mg.kg-1 fresh weight ± standard deviation) of the coumarins and the furanocoumarins in the grapefruits peel and pulp extracts.** The mention « Traces » is used for compounds that could be detected but not quantitated (3 < S/N < 10) while « Detected » is used when 5-geranyloxy-7-methoxycoumarin was identified in the samples.

| | | Washington Navel sweet orange | | Shamouti sweet orange | | Hamlin sweet orange | | Pineapple sweet orange | |
|---|---|---|---|---|---|---|---|---|---|
| | | Peel | Pulp | Peel | Pulp | Peel | Pulp | Peel | Pulp |
| | **Coumarins** | | | | | | | | |
| | Umbelliferone | | | | | | | | |
| | Osthol | Traces | 0.17 ± 0.01 | 0.14 ± 0.02 | Traces | 0.22 ± 0.04 | 0.20 ± 0.03 | | |
| | Aurapten | | | | | | | | |
| | Epoxyaurapten | | | | | | | | |
| | Limettin | 0.92 ± 0.13 | Traces | | | | | 6.15 ± 0.71 | 1.18 ± 0.18 |
| | 5-geranyloxy-7-methoxycoumarin | | | | | | | | |
| | **Furanocoumarins** | | | | | | | | |
| | Psoralen | | | | | | | | |
| Bergapten cluster | Bergaptol | | | | | | | | |
| | Bergapten | Traces | | | | | | 4.26 ± 0.40 | |
| | Isoimperatorin | | | | | | | | |
| | Oxypeucedanin | 0.73 ± 0.16 | 0.63 ± 0.10 | 0.41 ± 0.07 | 0.33 ± 0.01 | 0.53 ± 0.08 | 0.26 ± 0.03 | Traces | Traces |
| | Oxypeucedanin hydrate | | | | | | | | |
| | Bergamottin | | | | | | | | |
| | Epoxybergamottin | | | | | | | | |
| | 6',7'-dihydroxybergamottin | | | | | | | | |
| Xanthotoxin cluster | Xanthotoxol | | | | | | | | |
| | Xanthotoxin | | | | | | | | |
| | Imperatorin | | | | | | | | |
| | Heraclenin | | | | | | | | |
| | Heraclenol | | | | | | | | |
| | 8-geranyloxypsoralen | | | | | | | | |
| Isopimpinellin cluster | Isopimpinellin | | | | | | | 2.29 ± 0.56 | 0.58 ± 0.08 |
| | Phellopterin | | | | | | | | |
| | Byakangelicol | | | | | | | | |
| | Byakangelicin | | | | | | | | |
| | Cnidilin | | | | | | | | |
| | Cnidicin | | | | | | | | |
| TOTAL ($mg.kg^{-1}$) | | 1.65 | 0.80 | 0.55 | 0.33 | 0.75 | 0.46 | 12.70 | 1.76 |

**Table F. Concentration (in mg.kg-1 fresh weight ± standard deviation) of the coumarins and the furanocoumarins in the sweet oranges peel and pulp extracts.** The mention « Traces » is used for compounds that could be detected but not quantitated (3 < S/N < 10) while « Detected » is used when 5-geranyloxy-7-methoxycoumarin was identified in the samples.

| | | Maroc sour Orange | | Bouquetier de Nice sour orange | | Granito sour orange | | Chinotto sour orange | |
|---|---|---|---|---|---|---|---|---|---|
| | | Peel | Pulp | Peel | Pulp | Peel | Pulp | Peel | Pulp |
| | **Coumarins** | | | | | | | | |
| | Umbelliferone | | | | | | | | |
| | Osthol | 14.89 ± 2.83 | 0.79 ± 0.18 | 21.22 ± 4.97 | 1.77 ± 0.32 | 19.96 ± 2.93 | 0.96 ± 0.36 | 11.29 ± 3.84 | 1.91 ± 0.51 |
| | Aurapten | | | | | | | | |
| | Epoxyaurapten | | 2.89 ± 0.56 | | 9.97 ± 4.07 | | | | 3.17 ± 0.48 |
| | Limettin | | | | | | | | |
| | 5-geranyloxy-7-methoxycoumarin | Detected | Detected | Detected | Detected | Detected | Detected | Detected | Detected |
| | **Furanocoumarins** | | | | | | | | |
| | Psoralen | | Traces | | 0.35 ± 0.12 | | 0.16 ± 0.02 | | Traces |
| Bergapten cluster | Bergaptol | | 4.77 ± 1.42 | | 6.72 ± 1.76 | | 5.67 ± 2.22 | | |
| | Bergapten | 17.87 ± 5.77 | 39.81 ± 14.23 | 21.75 ± 5.28 | 107.76 ± 46.02 | 41.92 ± 2.29 | 79.08 ± 34.97 | 15.43 ± 6.90 | 75.48 ± 20.37 |
| | Isoimperatorin | 2.79 ± 0.38 | | 3.85 ± 0.72 | Traces | 7.65 ± 0.66 | Traces | 2.61 ± 0.69 | Traces |
| | Oxypeucedanin | 0.46 ± 0.12 | 0.28 ± 0.07 | 0.50 ± 0.02 | 0.28 ± 0.02 | 0.68 ± 0.04 | 0.70 ± 0.05 | 0.45 ± 0.06 | 0.22 ± 0.01 |
| | Oxypeucedanin hydrate | | | | | | | | |
| | Bergamottin | 0.35 ± 0.11 | 1.68 ± 0.42 | 0.69 ± 0.12 | 2.08 ± 0.68 | 1.08 ± 0.23 | 2.98 ± 0.45 | 0.59 ± 0.25 | 0.18 ± 0.11 |
| | Epoxybergamottin | 22.05 ± 5.43 | 7.57 ± 1.19 | 27.82 ± 8.59 | 7.32 ± 2.80 | 48.09 ± 7.84 | 11.40 ± 2.20 | 13.29 ± 2.23 | |
| | 6',7'-dihydroxybergamottin | 19.56 ± 3.76 | 11.26 ± 4.47 | 34.71 ± 9.09 | 23.29 ± 11.26 | 45.90 ± 4.72 | 20.96 ± 2.37 | 38.43 ± 15.78 | 10.68 ± 2.86 |
| Xanthotoxin cluster | Xanthotoxol | | | | | | | | |
| | Xanthotoxin | | | | | | | | |
| | Imperatorin | | Traces | | Traces | | | | Traces |
| | Heraclenin | | | | | | | | |
| | Heraclenol | | | | | | | | |
| | 8-geranyloxypsoralen | | | | | | | | |
| Isopimpinellin cluster | Isopimpinellin | Traces | Traces | Traces | Traces | Traces | Traces | Traces | Traces |
| | Phellopterin | | | | | | | | |
| | Byakangelicol | | | | | | | | |
| | Byakangelicin | | | | | | | | |
| | Cnidilin | | | | | | | | |
| | Cnidicin | | | | | | | | |
| | TOTAL ($mg.kg^{-1}$) | 77.97 | 69.05 | 110.54 | 159.54 | 165.28 | 121.91 | 82.09 | 91.64 |

**Table G. Concentration (in mg.kg-1 fresh weight ± standard deviation) of the coumarins and the furanocoumarins in the sour oranges peel and pulp extracts.** The mention « Traces » is used for compounds that could be detected but not quantitated (3 < S/N < 10) while « Detected » is used when 5-geranyloxy-7-methoxycoumarin was identified in the samples.

| | | Commune clementine | | Murcott tangor | | Bendiguangju | | Fortune mandarin | |
|---|---|---|---|---|---|---|---|---|---|
| | | Peel | Pulp | Peel | Pulp | Peel | Pulp | Peel | Pulp |
| | **Coumarins** | | | | | | | | |
| | Umbelliferone | 0.81 ± 0.19 | | | | | | | |
| | Osthol | | | | | | | | |
| | Aurapten | 0.15 ± 0.03 | 0.14 ± 0.03 | | | | | 0.29 ± 0.03 | |
| | Epoxyaurapten | | | | | | | | |
| | Limettin | 3.16 ± 0.24 | 0.42 ± 0.05 | Traces | | | | Traces | |
| | 5-geranyloxy-7-methoxycoumarin | | | Detected | Detected | Detected | Detected | Detected | Detected |
| | **Furanocoumarins** | | | | | | | | |
| | Psoralen | | | | | | | | |
| Bergapten cluster | Bergaptol | | | | | | | | |
| | Bergapten | 3.15 ± 0.27 | 0.38 ± 0.03 | | | | | | |
| | Isoimperatorin | | | | | | | | |
| | Oxypeucedanin | 0.94 ± 0.19 | 0.15 ± 0.02 | 0.60 ± 0.03 | 0.36 ± 0.09 | 0.53 ± 0.08 | 0.28 ± 0.04 | 0.62 ± 0.08 | 0.33 ± 0.06 |
| | Oxypeucedanin hydrate | 0.94 ± 0.27 | 0.34 ± 0.04 | | | | | | |
| | Bergamottin | | | | | | | | |
| | Epoxybergamottin | | | | | | | | |
| | 6',7'-dihydroxybergamottin | | | | | | | | |
| Xanthotoxin cluster | Xanthotoxol | | | | | | | | |
| | Xanthotoxin | | | | | | | | |
| | Imperatorin | | | | | | | | |
| | Heraclenin | | | | | | | | |
| | Heraclenol | | | | | | | | |
| | 8-geranyloxypsoralen | | | | | | | | |
| Isopimpinellin cluster | Isopimpinellin | 1.40 ± 0.26 | 0.28 ± 0.01 | Traces | | | | Traces | |
| | Phellopterin | | | | | | | | |
| | Byakangelicol | | | | | | | | |
| | Byakangelicin | | | | | | | | |
| | Cnidilin | | | | | | | | |
| | Cnidicin | | | | | | | | |
| | TOTAL (mg.kg$^{-1}$) | 10.55 | 1.71 | 0.60 | 0.36 | 0.53 | 0.28 | 0.91 | 0.33 |

**Table H. Concentration (in mg.kg-1 fresh weight ± standard deviation) of the coumarins and the furanocoumarins in the small mandarin hybrids peel and pulp extracts.** The mention « Traces » is used for compounds that could be detected but not quantitated (3 < S/N < 10) while « Detected » is used when 5-geranyloxy-7-methoxycoumarin was identified in the samples.

| | | Eureka lemon | | Meyer lemon | | Rough lemon | | Volkamer lemon | |
|---|---|---|---|---|---|---|---|---|---|
| | | Peel | Pulp | Peel | Pulp | Peel | Pulp | Peel | Pulp |
| | **Coumarins** | | | | | | | | |
| | Umbelliferone | Traces | | | | | | | |
| | Osthol | Traces | Traces | Traces | | | | | |
| | Aurapten | | | | | 0.91 ± 0.43 | 0.14 ± 0.03 | 0.70 ± 0.16 | 0.23 ± 0.07 |
| | Epoxyaurapten | | | | | | | | |
| | Limettin | 24.19 ± 5.24 | | 36.38 ± 0.87 | Traces | 2.51 ± 0.39 | Traces | 4.54 ± 1.23 | 1.15 ± 0.29 |
| | 5-geranyloxy-7-methoxycoumarin | Detected | Detected | Detected | | Detected | Detected | | |
| | **Furanocoumarins** | | | | | | | | |
| | Psoralen | | | | | | | | |
| Bergapten cluster | Bergaptol | | | | | | | | |
| | Bergapten | Traces | Traces | Traces | | 2.45 ± 0.60 | 0.71 ± 0.20 | 2.35 ± 0.24 | 0.43 ± 0.13 |
| | Isoimperatorin | Traces | | 4.97 ± 1.41 | Traces | | | 1.29 ± 0.22 | |
| | Oxypeucedanin | 10.48 ± 3.60 | 0.28 ± 0.12 | 17.12 ± 5.22 | 0.38 ± 0.05 | 3.08 ± 0.15 | 0.34 ± 0.09 | 1.52 ± 0.23 | 0.24 ± 0.03 |
| | Oxypeucedanin hydrate | 21.91 ± 12.56 | Traces | 2.97 ± 0.69 | Traces | | | 1.49 ± 0.55 | 0.29 ± 0.07 |
| | Bergamottin | 2.22 ± 1.30 | 0.13 ± 0.10 | | | 0.63 ± 0.15 | 0.05 ± 0.02 | | |
| | Epoxybergamottin | | | | | | | | |
| | 6',7'-dihydroxybergamottin | | Traces | | | | | | Traces |
| Xanthotoxin cluster | Xanthotoxol | | | | | | | | |
| | Xanthotoxin | | | | | | | | |
| | Imperatorin | | | | | | | | |
| | Heraclenin | 1.57 ± 0.86 | | | | | | 0.80 ± 0.28 | Traces |
| | Heraclenol | Traces | | | | | | 4.16 ± 1.44 | |
| | 8-geranyloxypsoralen | 6.75 ± 4.27 | Traces | | | | | | |
| Isopimpinellin cluster | Isopimpinellin | 0.82 ± 0.29 | | 2.73 ± 0.64 | Traces | 1.92 ± 0.38 | 0.71 ± 0.24 | 1.57 ± 0.23 | 0.63 ± 0.13 |
| | Phellopterin | 4.57 ± 2.17 | | | | | | | |
| | Byakangelicol | 18.15 ± 7.39 | | 0.92 ± 0.20 | | 2.79 ± 0.63 | | 0.72 ± 0.14 | |
| | Byakangelicin | 36.64 ± 16.18 | | | | 20.91 ± 5.39 | | 14.11 ± 4.53 | |
| | Cnidilin | | | | Traces | | | | |
| | Cnidicin | Traces | | | | | | | |
| | TOTAL (mg.kg$^{-1}$) | 127.30 | 0.41 | 65.09 | 0.38 | 35.20 | 1.95 | 33.25 | 2.97 |

| | | Marrakech lime | | Rangpur lime | | Yellow Rangpur lime | |
|---|---|---|---|---|---|---|---|
| | | Peel | Pulp | Peel | Pulp | Peel | Pulp |
| | **Coumarins** | | | | | | |
| | Umbelliferone | | | | | | |
| | Osthol | | | | | | |
| | Aurapten | 0.50 ± 0.21 | | 17.90 ± 11.15 | 0.03 ± 0.00 | 0.92 ± 0.43 | Traces |
| | Epoxyaurapten | 24.46 ± 7.87 | | | | | |
| | Limettin | 4.40 ± 1.91 | | 47.32 ± 10.17 | 1.45 ± 0.47 | 19.44 ± 2.31 | 1.24 ± 0.16 |
| | 5-geranyloxy-7-methoxycoumarin | | | | | | |
| | **Furanocoumarins** | | | | | | |
| | Psoralen | 0.24 ± 0.05 | | | | | |
| Bergapten cluster | Bergaptol | | | | | | |
| | Bergapten | 1.40 ± 0.27 | | 5.25 ± 0.73 | 0.99 ± 0.14 | 2.51 ± 0.19 | 0.89 ± 0.21 |
| | Isoimperatorin | | | 2.61 ± 0.65 | | 1.22 ± 0.39 | |
| | Oxypeucedanin | 11.38 ± 4.72 | 0.21 ± 0.04 | 25.66 ± 7.49 | 0.13 ± 0.01 | 10.14 ± 2.29 | 0.10 ± 0.01 |
| | Oxypeucedanin hydrate | 1.72 ± 0.73 | | 19.93 ± 3.20 | Traces | 2.72 ± 0.72 | |
| | Bergamottin | | | | | | |
| | Epoxybergamottin | | | 2.86 ± 0.33 | | Traces | |
| | 6',7'-dihydroxybergamottin | | | | | | |
| Xanthotoxin cluster | Xanthotoxol | | | | | | |
| | Xanthotoxin | | | | | | |
| | Imperatorin | | | | | | |
| | Heraclenin | 3.50 ± 1.84 | | | | | |
| | Heraclenol | Traces | | | | | |
| | 8-geranyloxypsoralen | | | | | | |
| Isopimpinellin cluster | Isopimpinellin | 0.32 ± 0.10 | | 4.96 ± 0.71 | 0.49 ± 0.09 | 3.77 ± 0.56 | 0.55 ± 0.05 |
| | Phellopterin | Traces | | Traces | Traces | Traces | Traces |
| | Byakangelicol | 9.54 ± 2.08 | | 1.80 ± 0.42 | | 2.82 ± 0.15 | |
| | Byakangelicin | | | | | | |
| | Cnidilin | Traces | | Traces | Traces | Traces | Traces |
| | Cnidicin | | | | | | |
| | TOTAL (mg.kg$^{-1}$) | 57.46 | 0.21 | 128.29 | 3.09 | 43.54 | 2.78 |

**Tables I. Concentration (in mg.kg-1 fresh weight ± standard deviation) of the coumarins and the furanocoumarins in the lemons peel and pulp extracts.** The mention « Traces » is used for compounds that could be detected but not quantitated ($3 < S/N < 10$) while « Detected » is used when 5-geranyloxy-7-methoxycoumarin was identified in the samples.

| Cluster | Compound | Mexican lime Peel | Mexican lime Pulp | Giant Key lime Peel | Giant Key lime Pulp | Coppenrhad lime Peel | Coppenrhad lime Pulp | Excelsa Peel | Excelsa Pulp |
|---|---|---|---|---|---|---|---|---|---|
| | **Coumarins** | | | | | | | | |
| | Umbelliferone | 3.04 ± 1.15 | Traces | 1.38 ± 0.51 | Traces | 0.85 ± 0.12 | 0.49 ± 0.18 | | 0.24 ± 0.02 |
| | Osthol | | | 0.21 ± 0.07 | Traces | 0.15 ± 0.04 | Traces | | |
| | Aurapten | Traces | 0.33 ± 0.21 | Traces | 0.17 ± 0.07 | Traces | 0.22 ± 0.15 | 0.61 ± 0.18 | 0.09 ± 0.02 |
| | Epoxyaurapten | Traces | | | | | | Traces | |
| | Limettin | 47.74 ± 12.59 | 1.42 ± 0.22 | 15.21 ± 5.77 | Traces | 14.59 ± 6.67 | Traces | 8.06 ± 0.89 | 1.10 ± 0.10 |
| | 5-geranyloxy-7-methoxycoumarin | Detected | Detected | Detected | Detected | Detected | Detected | Detected | Detected |
| | **Furanocoumarins** | | | | | | | | |
| | Psoralen | | | | | | | | |
| Bergapten cluster | Bergaptol | | | | 2.17 ± 0.26 | | 0.67 ± 0.27 | 3.12 ± 1.30 | Traces |
| Bergapten cluster | Bergapten | 6.08 ± 3.11 | 0.63 ± 0.08 | 0.70 ± 0.32 | 0.30 ± 0.08 | 0.40 ± 0.02 | 0.15 ± 0.06 | 4.92 ± 1.00 | 0.72 ± 0.15 |
| Bergapten cluster | Isoimperatorin | 16.35 ± 3.09 | 1.11 ± 0.47 | 1.66 ± 0.72 | 0.92 ± 0.41 | Traces | | 12.90 ± 4.98 | Traces |
| Bergapten cluster | Oxypeucedanin | 56.89 ± 7.00 | 0.42 ± 0.11 | 21.71 ± 5.69 | 0.44 ± 0.07 | 16.59 ± 6.56 | 0.26 ± 0.05 | 81.05 ± 23.24 | 0.17 ± 0.03 |
| Bergapten cluster | Oxypeucedanin hydrate | 24.77 ± 9.92 | 11.95 ± 5.41 | 4.08 ± 2.12 | 2.43 ± 1.71 | 1.86 ± 0.27 | Traces | 34.81 ± 12.83 | 0.45 ± 0.20 |
| Bergapten cluster | Bergamottin | 16.05 ± 4.69 | 15.06 ± 7.70 | 44.62 ± 9.57 | 12.99 ± 4.58 | 23.97 ± 2.82 | 1.58 ± 1.14 | 29.04 ± 10.98 | 0.13 ± 0.06 |
| Bergapten cluster | Epoxybergamottin | Traces | Traces | | | | | 117.29 ± 33.15 | |
| Bergapten cluster | 6',7'-dihydroxybergamottin | | Traces | | | | | 37.10 ± 15.27 | Traces |
| Xanthotoxin cluster | Xanthotoxol | | 6.35 ± 1.05 | | 1.42 ± 0.21 | | Traces | | |
| Xanthotoxin cluster | Xanthotoxin | 4.84 ± 3.11 | 0.72 ± 0.43 | | 0.31 ± 0.12 | | | 0.20 ± 0.02 | |
| Xanthotoxin cluster | Imperatorin | 5.03 ± 2.01 | 6.64 ± 1.43 | Traces | 2.21 ± 0.76 | | | 4.33 ± 0.94 | |
| Xanthotoxin cluster | Heraclenin | 33.96 ± 2.75 | 0.97 ± 0.36 | 6.42 ± 2.68 | 0.72 ± 0.34 | Traces | Traces | 2.02 ± 0.12 | Traces |
| Xanthotoxin cluster | Heraclenol | 7.88 ± 3.71 | 9.93 ± 4.43 | | 2.04 ± 1.17 | | | Traces | |
| Xanthotoxin cluster | 8-geranyloxypsoralen | 48.08 ± 10.96 | 13.34 ± 4.74 | 24.66 ± 10.18 | 15.09 ± 6.79 | 16.10 ± 4.38 | 12.62 ± 7.50 | 11.55 ± 3.57 | Traces |
| Isopimpinellin cluster | Isopimpinellin | 50.76 ± 8.29 | 3.29 ± 1.46 | 24.68 ± 7.60 | 1.36 ± 0.64 | 1.34 ± 0.15 | 0.18 ± 0.10 | 3.09 ± 0.23 | 0.51 ± 0.08 |
| Isopimpinellin cluster | Phellopterin | 18.85 ± 6.74 | 1.35 ± 0.21 | 2.55 ± 0.77 | 1.29 ± 0.63 | 1.15 ± 0.33 | Traces | 10.82 ± 1.82 | Traces |
| Isopimpinellin cluster | Byakangelicol | 73.37 ± 7.21 | Traces | 23.33 ± 9.00 | Traces | 10.00 ± 1.87 | Traces | 41.63 ± 8.43 | Traces |
| Isopimpinellin cluster | Byakangelicin | Traces | 14.43 ± 4.47 | Traces | Traces | Traces | Traces | | |
| Isopimpinellin cluster | Cnidilin | 47.71 ± 7.84 | 2.80 ± 1.22 | 9.75 ± 3.44 | 2.09 ± 1.12 | 2.37 ± 0.97 | 0.17 ± 0.03 | | |
| Isopimpinellin cluster | Cnidicin | 4.83 ± 1.74 | 1.57 ± 0.41 | 1.04 ± 0.45 | Traces | Traces | Traces | 2.64 ± 0.49 | |
| | TOTAL ($mg.kg^{-1}$) | 466.23 | 92.31 | 182.00 | 45.95 | 89.37 | 16.34 | 405.18 | 3.41 |

| Cluster | Compound | Tahiti lime Peel | Tahiti lime Pulp | Bears lime Peel | Bears lime Pulp | Brazil sweet lime Peel | Brazil sweet lime Pulp | Palestine sweet lime Peel | Palestine sweet lime Pulp |
|---|---|---|---|---|---|---|---|---|---|
| | **Coumarins** | | | | | | | | |
| | Umbelliferone | | | | | Traces | | Traces | |
| | Osthol | | | | | | | Traces | Traces |
| | Aurapten | 1.17 ± 0.20 | 0.25 ± 0.08 | 1.20 ± 0.41 | 0.30 ± 0.05 | Traces | | Traces | |
| | Epoxyaurapten | | | | | | | | |
| | Limettin | 359.33 ± 147.68 | Traces | 140.60 ± 45.93 | Traces | 25.35 ± 10.13 | Traces | 29.18 ± 4.70 | Traces |
| | 5-geranyloxy-7-methoxycoumarin | Detected | Detected | Detected | Detected | Detected | Detected | | Detected |
| | **Furanocoumarins** | | | | | | | | |
| | Psoralen | 0.33 ± 0.13 | | Traces | | | | | |
| Bergapten cluster | Bergaptol | | | | | | | | |
| Bergapten cluster | Bergapten | 288.49 ± 30.74 | 1.71 ± 0.49 | 173.22 ± 54.20 | 1.80 ± 1.28 | 1.26 ± 0.22 | | Traces | |
| Bergapten cluster | Isoimperatorin | 1.63 ± 0.33 | Traces | 3.17 ± 0.69 | Traces | Traces | | Traces | |
| Bergapten cluster | Oxypeucedanin | 55.23 ± 11.68 | 1.09 ± 0.42 | 67.48 ± 10.88 | 1.00 ± 0.43 | 18.77 ± 6.47 | 0.25 ± 0.02 | 13.96 ± 4.72 | 0.32 ± 0.05 |
| Bergapten cluster | Oxypeucedanin hydrate | 11.69 ± 4.20 | 1.15 ± 0.45 | 11.86 ± 3.54 | 1.08 ± 0.45 | 3.10 ± 0.37 | | 2.74 ± 0.83 | |
| Bergapten cluster | Bergamottin | 44.27 ± 14.75 | 19.76 ± 7.58 | 29.83 ± 21.82 | 9.75 ± 5.83 | Traces | Traces | | |
| Bergapten cluster | Epoxybergamottin | | | | | | | | |
| Bergapten cluster | 6',7'-dihydroxybergamottin | | | | | | | | |
| Xanthotoxin cluster | Xanthotoxol | | | | | | | | |
| Xanthotoxin cluster | Xanthotoxin | 4.61 ± 1.57 | | 4.07 ± 0.46 | | | | | |
| Xanthotoxin cluster | Imperatorin | | | | | | | | |
| Xanthotoxin cluster | Heraclenin | 5.64 ± 1.21 | | 7.72 ± 0.50 | | 1.77 ± 0.77 | | 1.63 ± 0.73 | |
| Xanthotoxin cluster | Heraclenol | | | | | Traces | | | |
| Xanthotoxin cluster | 8-geranyloxypsoralen | 21.58 ± 4.21 | 16.41 ± 7.77 | 23.97 ± 2.60 | 8.10 ± 4.02 | | | | |
| Isopimpinellin cluster | Isopimpinellin | 87.20 ± 11.92 | 2.92 ± 1.21 | 57.16 ± 10.03 | 2.92 ± 1.35 | 1.22 ± 0.09 | Traces | 1.28 ± 0.17 | Traces |
| Isopimpinellin cluster | Phellopterin | 2.83 ± 0.66 | Traces | 3.28 ± 0.16 | 1.74 ± 0.97 | | | Traces | |
| Isopimpinellin cluster | Byakangelicol | 17.25 ± 3.76 | | 13.80 ± 2.56 | | 6.67 ± 1.70 | | 7.26 ± 1.56 | |
| Isopimpinellin cluster | Byakangelicin | | | | | | | | |
| Isopimpinellin cluster | Cnidilin | 1.91 ± 0.28 | 0.80 ± 0.22 | 3.51 ± 0.37 | Traces | | | | |
| Isopimpinellin cluster | Cnidicin | | Traces | | Traces | | | | |
| | TOTAL ($mg.kg^{-1}$) | 903.16 | 44.09 | 540.87 | 26.69 | 58.14 | 0.25 | 56.05 | 0.32 |

| Cluster | Compound | Alemow Peel | Alemow Pulp |
|---|---|---|---|
| | **Coumarins** | | |
| | Umbelliferone | 1.35 ± 0.37 | 0.51 ± 0.25 |
| | Osthol | | |
| | Aurapten | 51.64 ± 28.60 | 14.47 ± 4.12 |
| | Epoxyaurapten | 48.84 ± 11.80 | Traces |
| | Limettin | 84.71 ± 25.73 | 1.94 ± 0.23 |
| | 5-geranyloxy-7-methoxycoumarin | Detected | Detected |
| | **Furanocoumarins** | | |
| | Psoralen | | |
| Bergapten cluster | Bergaptol | | |
| Bergapten cluster | Bergapten | 10.07 ± 2.43 | 1.04 ± 0.15 |
| Bergapten cluster | Isoimperatorin | 36.78 ± 6.87 | Traces |
| Bergapten cluster | Oxypeucedanin | 81.44 ± 17.26 | |
| Bergapten cluster | Oxypeucedanin hydrate | 49.72 ± 13.98 | 0.50 ± 0.02 |
| Bergapten cluster | Bergamottin | 4.73 ± 2.70 | Traces |
| Bergapten cluster | Epoxybergamottin | 10.67 ± 1.52 | |
| Bergapten cluster | 6',7'-dihydroxybergamottin | 38.17 ± 6.42 | Traces |
| Xanthotoxin cluster | Xanthotoxol | | 1.00 ± 0.34 |
| Xanthotoxin cluster | Xanthotoxin | | |
| Xanthotoxin cluster | Imperatorin | 23.92 ± 6.40 | 2.49 ± 0.95 |
| Xanthotoxin cluster | Heraclenin | 61.43 ± 8.20 | 1.02 ± 0.44 |
| Xanthotoxin cluster | Heraclenol | 32.96 ± 5.69 | 1.73 ± 0.62 |
| Xanthotoxin cluster | 8-geranyloxypsoralen | 15.18 ± 6.84 | 3.18 ± 0.82 |
| Isopimpinellin cluster | Isopimpinellin | 4.85 ± 0.45 | 0.70 ± 0.12 |
| Isopimpinellin cluster | Phellopterin | 40.98 ± 5.30 | 4.50 ± 1.31 |
| Isopimpinellin cluster | Byakangelicol | 72.00 ± 12.56 | 1.79 ± 0.72 |
| Isopimpinellin cluster | Byakangelicin | 33.13 ± 8.43 | 13.46 ± 3.68 |
| Isopimpinellin cluster | Cnidilin | 13.89 ± 1.10 | |
| Isopimpinellin cluster | Cnidicin | 7.14 ± 1.05 | Traces |
| | TOTAL ($mg.kg^{-1}$) | 723.60 | 48.33 |

**Tables J. Concentration (in mg.kg-1 fresh weight ± standard deviation) of the coumarins and the furanocoumarins in the limes peel and pulp extracts.** The mention « Traces » is used for compounds that could be detected but not quantitated (3 < S/N < 10) while « Detected » is used when 5-geranyloxy-7-methoxycoumarin was identified in the samples.

| | | Castagnaro bergamot | |
|---|---|---|---|
| | | Peel | Pulp |
| | **Coumarins** | | |
| | Umbelliferone | | |
| | Osthol | 0.39 ± 0.10 | 0.10 ± 0.02 |
| | Aurapten | 0.20 ± 0.09 | 0.19 ± 0.09 |
| | Epoxyaurapten | 10.72 ± 1.81 | |
| | Limettin | 25.69 ± 3.66 | 1.30 ± 0.46 |
| | 5-geranyloxy-7-methoxycoumarin | Detected | Detected |
| | **Furanocoumarins** | | |
| | Psoralen | 0.47 ± 0.13 | 0.57 ± 0.49 |
| Bergapten cluster | Bergaptol | | 8.57 ± 1.02 |
| | Bergapten | 388.21 ± 105.05 | 51.56 ± 43.53 |
| | Isoimperatorin | Traces | 0.88 ± 0.17 |
| | Oxypeucedanin | 0.80 ± 0.52 | 0.25 ± 0.01 |
| | Oxypeucedanin hydrate | | |
| | Bergamottin | 54.70 ± 38.91 | 49.67 ± 31.26 |
| | Epoxybergamottin | | |
| | 6',7'-dihydroxybergamottin | | |
| Xanthotoxin cluster | Xanthotoxol | | |
| | Xanthotoxin | | |
| | Imperatorin | | |
| | Heraclenin | | |
| | Heraclenol | | Traces |
| | 8-geranyloxypsoralen | | Traces |
| Isopimpinellin cluster | Isopimpinellin | 2.57 ± 0.27 | 0.30 ± 0.15 |
| | Phellopterin | | |
| | Byakangelicol | | |
| | Byakangelicin | | |
| | Cnidilin | | |
| | Cnidicin | | |
| | TOTAL ($mg.kg^{-1}$) | 483.75 | 113.39 |

**Table K. Concentration (in mg.kg-1 fresh weight ± standard deviation) of the coumarins and the furanocoumarins in the bergamot peel and pulp extracts.** The mention « Traces » is used for compounds that could be detected but not quantitated (3 < S/N < 10) while « Detected » is used when 5-geranyloxy-7-methoxycoumarin was identified in the samples.

| | | Mountain | | Yuzu | | Khasi | | Nasnaran | |
|---|---|---|---|---|---|---|---|---|---|
| | | Peel | Pulp | Peel | Pulp | Peel | Pulp | Peel | Pulp |
| | **Coumarins** | | | | | | | | |
| | Umbelliferone | | | | | Traces | | 1.76 ± 0.40 | |
| | Osthol | | | 1.88 ± 0.44 | | | | | |
| | Aurapten | 0.36 ± 0.06 | 0.22 ± 0.05 | 20.64 ± 11.65 | 3.60 ± 1.21 | 0.14 ± 0.03 | 0.05 ± 0.02 | 1.39 ± 0.34 | 0.13 ± 0.04 |
| | Epoxyaurapten | | | 17.54 ± 2.43 | | | | | |
| | Limettin | 4.17 ± 1.06 | 0.93 ± 0.37 | 4.59 ± 1.08 | | 5.04 ± 0.25 | 0.58 ± 0.10 | 12.58 ± 3.13 | 1.30 ± 0.26 |
| | 5-geranyloxy-7-methoxycoumarin | Detected | | Detected | Detected | Detected | Detected | Detected | Detected |
| | **Furanocoumarins** | | | | | | | | |
| | Psoralen | | | | | | | | |
| Bergapten cluster | Bergaptol | | | | | | Traces | 17.66 ± 7.38 | 6.62 ± 2.07 |
| | Bergapten | 3.72 ± 0.95 | 0.70 ± 0.20 | 21.91 ± 2.42 | Traces | 3.53 ± 0.11 | 0.44 ± 0.06 | 44.11 ± 10.19 | 1.56 ± 0.48 |
| | Isoimperatorin | Traces | Traces | | | Traces | | 40.54 ± 8.66 | 2.92 ± 1.01 |
| | Oxypeucedanin | 2.37 ± 0.34 | 0.08 ± 0.01 | 0.39 ± 0.22 | 0.30 ± 0.08 | 1.34 ± 0.20 | 0.13 ± 0.01 | 278.71 ± 74.61 | 6.94 ± 2.40 |
| | Oxypeucedanin hydrate | 0.32 ± 0.08 | 0.20 ± 0.04 | | | Traces | | 135.44 ± 36.17 | 14.51 ± 3.34 |
| | Bergamottin | Traces | | 0.69 ± 0.10 | Traces | Traces | | 55.94 ± 21.99 | 10.26 ± 3.71 |
| | Epoxybergamottin | | | | | | | 61.01 ± 15.30 | 4.96 ± 1.68 |
| | 6',7'-dihydroxybergamottin | | | | | | Traces | 101.36 ± 16.35 | 12.82 ± 5.90 |
| Xanthotoxin cluster | Xanthotoxol | | | | | | Traces | | |
| | Xanthotoxin | | | | | 1.39 ± 0.67 | 0.21 ± 0.11 | Traces | |
| | Imperatorin | | | | | | | | |
| | Heraclenin | | | | | 6.13 ± 1.60 | Traces | | |
| | Heraclenol | | | | | Traces | Traces | | |
| | 8-geranyloxypsoralen | | | | | 15.62 ± 1.75 | 1.84 ± 0.76 | | 2.26 ± 0.75 |
| Isopimpinellin cluster | Isopimpinellin | 2.33 ± 0.39 | 0.61 ± 0.21 | 2.61 ± 0.67 | 0.46 ± 0.19 | 13.14 ± 3.02 | 0.39 ± 0.03 | 6.70 ± 1.28 | 0.88 ± 0.12 |
| | Phellopterin | | | | | | | | |
| | Byakangelicol | | | | | 4.05 ± 0.74 | | Traces | |
| | Byakangelicin | | | | | | | | |
| | Cnidilin | | | | | Traces | Traces | | |
| | Cnidicin | | | | | | | | |
| | TOTAL (mg.kg$^{-1}$) | 13.27 | 2.74 | 70.25 | 4.36 | 50.38 | 3.64 | 757.20 | 65.16 |

| | | Mandarin x citron hybrid | |
|---|---|---|---|
| | | Peel | Pulp |
| | **Coumarins** | | |
| | Umbelliferone | | |
| | Osthol | | |
| | Aurapten | 0.80 ± 0.22 | 0.08 ± 0.02 |
| | Epoxyaurapten | | |
| | Limettin | 2.79 ± 0.08 | 0.48 ± 0.04 |
| | 5-geranyloxy-7-methoxycoumarin | Detected | Detected |
| | **Furanocoumarins** | | |
| | Psoralen | | |
| Bergapten cluster | Bergaptol | | |
| | Bergapten | 2.38 ± 0.26 | 0.46 ± 0.07 |
| | Isoimperatorin | 1.00 ± 0.13 | |
| | Oxypeucedanin | 2.14 ± 0.44 | 0.12 ± 0.01 |
| | Oxypeucedanin hydrate | | |
| | Bergamottin | Traces | |
| | Epoxybergamottin | Traces | |
| | 6',7'-dihydroxybergamottin | | |
| Xanthotoxin cluster | Xanthotoxol | | |
| | Xanthotoxin | | |
| | Imperatorin | | |
| | Heraclenin | | |
| | Heraclenol | Traces | |
| | 8-geranyloxypsoralen | | |
| Isopimpinellin cluster | Isopimpinellin | 1.45 ± 0.20 | 0.36 ± 0.06 |
| | Phellopterin | | |
| | Byakangelicol | 1.00 ± 0.35 | |
| | Byakangelicin | Traces | |
| | Cnidilin | | |
| | Cnidicin | | |
| | TOTAL (mg.kg$^{-1}$) | 11.56 | 1.50 |

**Tables L. Concentration (in mg.kg-1 fresh weight ± standard deviation) of the coumarins and the furanocoumarins in the unknown origin hybrids peel and pulp extracts.** The mention « Traces » is used for compounds that could be detected but not quantitated (3 < S/N < 10) while « Detected » is used when 5-geranyloxy-7-methoxycoumarin was identified in the samples.
