# Supplementary material for: The Distribution of Coumarins and Furanocoumarins in Citrus Species Closely Matches Citrus Phylogeny and Reflects the Organization of Biosynthetic Pathways
Source: PLoS One. 2015 Nov 11;10(11):e0142757. doi: 10.1371/journal.pone.0142757 (PMC4641707; doi:10.1371/journal.pone.0142757)
Supplement: S1 Table — (PDF) [file pone.0142757.s004.pdf]

S1 Table. List of the 61 citrus species investigated in the study and their phylogenetic constitution.

Horticultural group	Common name of ancestral species	SRA number	Swingle's classification system	Tanaka's classification system	Phylogenetic constitution	References
Papeda	Micrantha	-	*C. micrantha* Wester	*C. micrantha* Wester	*C. micrantha*	(8, 10, 12, 14)
	Combava	630	*C. hystrix* D.C.	*C. hystrix* D.C.	*C. micrantha*	
Citron	Corsican citron	613	*C. medica* L.	*C. medica* L.	*C. medica*	(8, 10, 12, 14)
	Etrog citron	709	*C. medica* L.	*C. medica* L.	*C. medica*	
	Buddha's Hand citron	640	*C. medica* L.	*C. medica* L.	*C. medica*	
Pummelo	Deep Red pummelo	757	*C. maxima* (Burm.) Merr.	*C. maxima* (Burm.) Merr.	*C. maxima*	(8, 10, 12, 14)
	Reinking pummelo	323	*C. maxima* (Burm.) Merr.	*C. maxima* (Burm.) Merr.	*C. maxima*	
	Tahiti pummelo	727	*C. maxima* (Burm.) Merr.	*C. maxima* (Burm.) Merr.	*C. maxima*	
	Chandler pummelo	608	*C. maxima* (Burm.) Merr.	*C. maxima* (Burm.) Merr.	*C. maxima*	
	Sans pépin pummelo	710	*C. maxima* (Burm.) Merr.	*C. maxima* (Burm.) Merr.	*C. maxima*	
	Kao Pan pummelo	321	*C. maxima* (Burm.) Merr.	*C. maxima* (Burm.) Merr.	*C. maxima*	
	Pink pummelo	322	*C. maxima* (Burm.) Merr.	*C. maxima* (Burm.) Merr.	*C. maxima*	
Mandarin	Willowleaf mandarin	133	*C. reticulata* Blanco	*C. deliciosa* Ten.	*C. reticulata*	(8, 10, 12, 14)
	Fuzhu mandarin	599	*C. reticulata* Blanco	*C. erythrosa* Hort. ex Tan.	*C. reticulata*	
	San Hu Hong Chu mandarin	769	*C. reticulata* Blanco	*C. erythrosa* Hort. ex Tan.	*C. reticulata*	
	Nan Feng Mi Ju mandarin	839	*C. reticulata* Blanco	*C. kinokuni* Hort. ex Tan.	*C. reticulata*	
	Beauty mandarin	261	*C. reticulata* Blanco	*C. reticulata* Blanco	*C. reticulata*	
	Dancy mandarin	552	*C. reticulata* Blanco	*C. tangerina* Hort. ex Tan.	*C. reticulata*	
	Wase satsuma	230	*C. reticulata* Blanco	*C. unshiu* Marc.	*C. reticulata*	
	Owari satsuma	221	*C. reticulata* Blanco	*C. unshiu* Marc.	*C. reticulata*	
	Sunki mandarin	705	*C. reticulata* Blanco var. austera Swing.	*C. sunki* (Hayata) Hort. ex Tan.	*C. reticulata* var austera	
	Shekwasha mandarin	982	*C. reticulata* Blanco hybrid	*C. depressa* Hayata	*C. reticulata* var austera	
	Cleopatra mandarin	948	*C. reticulata* Blanco var. austera Swing.	*C. reshni* Hort. ex Tan.	*C. reticulata* var austera	
**Horticultural group**	**Common name of secondary species**	**SRA number**	**Swingle's classification system**	**Tanaka's classification system**	**Phylogenetic constitution**	
Small mandarin hybrid	Commune Clementine	92	*C. reticulata* × *C. sinensis*	*C. clementina* Hort.ex Tan.	*C. reticulata* x ((*C. maxima* x *C. reticulata*) x *C. reticulata*)	(49)
	Murcott tangor	601	*C. reticulata* × *C. sinensis ?*	*C. reticulata* × *C. sinensis ?*	*C. reticulata* x ((*C. maxima* x *C. reticulata*) x *C. reticulata*) ?	
	Bendiguangju	578	*C. reticulata* Blanco	*C. nobilis* Lour.	?	-
	Fortune mandarin	31	(*C. reticulata* × *C. sinensis*) × *C. tangerina*	*C. clementina* × *C. tangerina*	((*C. reticulata* x ((*C. maxima* x *C. reticulata*) x *C. reticulata*))) x (*C. reticulata* x (*C. maxima* x ((*C. maxima* x *C. reticulata*) x *C. reticulata*)))	(49)
Sweet orange	Washington Navel sweet orange	217	*C. sinensis* (L.) Osbeck	*C. sinensis* (L.) Osbeck	(*C. maxima* x *C. reticulata*) x *C. reticulata*	(8, 12, 14, 49)
	Shamouti sweet orange	299	*C. sinensis* (L.) Osbeck	*C. sinensis* (L.) Osbeck	(*C. maxima* x *C. reticulata*) x *C. reticulata*	
	Hamlin sweet orange	41	*C. sinensis* (L.) Osbeck	*C. sinensis* (L.) Osbeck	(*C. maxima* x *C. reticulata*) x *C. reticulata*	
	Pineapple sweet orange	42	*C. sinensis* (L.) Osbeck	*C. sinensis* (L.) Osbeck	(*C. maxima* x *C. reticulata*) x *C. reticulata*	
Grapefruit	Duncan grapefruit	470	*C. paradisi* Macf.	*C. paradisi* Macf.	*C. maxima* x ((*C. maxima* x *C. reticulata*) x *C. reticulata*)	(8, 12, 14, 49)
	Marsh grapefruit	284	*C. paradisi* Macf.	*C. paradisi* Macf.	*C. maxima* x ((*C. maxima* x *C. reticulata*) x *C. reticulata*)	
	Star Ruby grapefruit	293	*C. paradisi* Macf.	*C. paradisi* Macf.	*C. maxima* x ((*C. maxima* x *C. reticulata*) x *C. reticulata*)	
Sour orange	Maroc sour orange	122	*C. aurantium* L.	*C. aurantium* L.	*C. maxima* x *C. reticulata*	(8, 12, 14, 49)
	Bouquetier de Nice sour orange	952	*C. aurantium* L.	*C. aurantium* L.	*C. maxima* x *C. reticulata*	
	Granito sour orange	870	*C. aurantium* L.	*C. aurantium* L.	*C. maxima* x *C. reticulata*	
	Chinotto sour orange	752	*C. aurantium* L.	*C. myrtifolia* Raf.	*C. maxima* x *C. reticulata*	
Lemon	Eureka lemon	2	*C. limon* (L.) Burm. f.	*C. limon* (L.) Burm. f.	(*C. maxima* x *C. reticulata*) x *C. medica*	(49)
	Meyer lemon	292	*C. limon* (L.) Burm. f.	*C. limon* (L.) Burm. f.	(*C. maxima* x *C. reticulata*) x *C. medica* **or** ((*C. maxima* x *C. reticulata*)x *C. reticulata*) x *C. medica*	
	Rough lemon	778	*C. limon* (L.) Burm. f.	*C. jambhiri* Lush.	*C. reticulata* x *C. medica*	
	Volkamer lemon	729	*C. limon* (L.) Burm. f.	*C. limonia* Osbeck	*C. reticulata* x *C. medica*	
	Marrakech lime	829	*C. limon* (L.) Burm. f.	*C. limetta* Risso	(*C. maxima* x *C. reticulata*) x *C. medica*	
	Rangpur lime	110050	*C. limon* (L.) Burm. f.	*C. limonia* Osbeck	*C. reticulata* x *C. medica*	
	Yellow Rangpur lime	944	*C. limon* (L.) Burm. f.	*C. limonia* Osbeck	*C. reticulata* x *C. medica*	
Lime	Mexican lime	140	*C. aurantifolia* (Christm.) Swing.	*C. aurantifolia* (Christm.) Swing.	*C. micrantha* x *C. medica*	(14)
	Giant Key lime	785	*C. aurantifolia* (Christm.) Swing.	*C. aurantifolia* (Christm.) Swing.	*C. micrantha* x *C. medica*	
	Coppenrhad lime	838	*C. aurantifolia* (Christm.) Swing.	*C. aurantifolia* (Christm.) Swing.	2 (*C. micrantha* x *C. medica*) x *C. medica*	(49)
	Excelsa	110234	*C. aurantifolia* (Christm.) Swing.	*C. excelsa* Wester	*C. micrantha* x *C. medica*	(14)
	Tahiti lime	58	*C. aurantifolia* (Christm.) Swing.	*C. latifolia* (Yu. Tan.) Tan.	((*C. maxima* x *C. reticulata*) x *C. medica*) x 2 (*C. micrantha* x *C. medica*)	(49)
	Bears lime	616	*C. aurantifolia* (Christm.) Swing.	*C. latifolia* (Yu. Tan.) Tan.	((*C. maxima* x *C. reticulata*) x *C. medica*) x 2 (*C. micrantha* x *C. medica*)	
	Brazil sweet lime	697	*C. aurantifolia* (Christm.) Swing.	*C. limettioides* Tan.	(*C. maxima* x *C. reticulata*) x *C. medica*	(14, 49)
	Palestine sweet lime	802	*C. aurantifolia* (Christm.) Swing.	*C. limettioides* Tan.	(*C. maxima* x *C. reticulata*) x *C. medica*	
	Alemow	779	*C. aurantifolia* (Christm.) Swing.	*C. macrophylla* Wester	*C. micrantha* x *C. medica*	(14)
Bergamot	Castagnaro Bergamot	612	*C. aurantifolia* var Bergamia	*C. bergamia* Risso and Poit.	((*C. maxima* x *C. reticulata*) x *C. medica*) x (*C. maxima* x *C. reticulata*)	(49)
Amblycarpa	Nasnaran mandarin	896	*C. reticulata* Blanco hybrid	*C. amblycarpa* (Hassk.) Ochse.	*C. micrantha* x *C. reticulata*	(14)
Mandarin × citron hybrid	Mandarin × citron hybrid	1057	*C. reticulata* Blanco var. austera Swing. x *C. medica* L.	*C. reticulata* Blanco var. austera Swing. x *C. medica* L.	*C. reticulata* x *C. medica*	(49)
Ichang	Ichang	687	*C. ichangensis* Swing.	*C. ichangensis* Swing.	?	-
Halimii	Mountain	110302	*C. halimii* B.C. Stone (Tan. Class.)	*C. halimii* B.C. Stone	?	-
Yuzu	Yuzu	846	*C. ichang austera* ex. Tan. hybrid	*C. junos* Siebold	?	-
Latipes	Khasi	844	*C. latipes* (Swing.) Tan.	*C. latipes* (Swing.) Tan.	?	-
