# Supplementary material for: The Distribution of Coumarins and Furanocoumarins in Citrus Species Closely Matches Citrus Phylogeny and Reflects the Organization of Biosynthetic Pathways
Source: PLoS One. 2015 Nov 11;10(11):e0142757. doi: 10.1371/journal.pone.0142757 (PMC4641707; doi:10.1371/journal.pone.0142757)
Supplement: S2 Table — (PDF) [file pone.0142757.s005.pdf]

	Limit of detection (mg/kg)	Limit of quantitation (mg/kg)	Specificity	
			Equation	r^2
**Coumarins**				
Umbelliferone	0,01	0,03	-	-
Osthol	0,00	0,01	-	-
Aurapten	0,01	0,03	y = 0,0008 x	0,9969
Epoxyaurapten	0,09	0,30	-	-
Limettin	0,01	0,02	-	-
5-geranyloxy-7-methoxycoumarin	0,00	0,01	-	-
**Furanocoumarins**				
Psoralen	0,01	0,03	-	-
Bergaptol	0,13	0,43	-	-
Bergapten	0,02	0,06	y = 0,0067 x	0,9990
Isoimperatorin	0,09	0,29	-	-
Oxypeucedanin	0,02	0,05	y = 0,0056 x	0,9989
Oxypeucedanin hydrate	0,01	0,04	y = 0,0658 x	0,9951
Bergamottin	0,01	0,02	-	-
Epoxybergamottin	0,04	0,12	-	-
6',7'-dihydroxybergamottin	0,62	2,06	-	-
Xanthotoxol	0,01	0,04	-	-
Xanthotoxin	0,01	0,03	-	-
Imperatorin	0,05	0,17	-	-
Heraclenin	0,05	0,15	-	-
Heraclenol	0,08	0,28	-	-
8-geranyloxypsoralen	0,32	1,07	y = 0,0935 x	0,9959
Isopimpinellin	0,02	0,08	y = 0,0168 x	0,9919
Phellopterin	0,17	0,57	-	-
Byakangelicol	0,01	0,02	-	-
Byakangelicin	1,28	4,26	-	-
Cnidilin	0,02	0,08	-	-
Cnidicin	0,31	1,03	-	-

S2 Table. Limits of detection and quantitation of the coumarins and furanocoumarins in the citrus pulp and specificity (equation and coefficient of determination, r^2) of the UPLC-MS method.
